# Unravelling the analgesic effects of perioperative magnesium in general abdominal surgery: a systematic review and meta-analysis of randomized controlled trials

**DOI:** 10.1016/j.bjane.2024.844524

**Published:** 2024-06-05

**Authors:** Yasin Avci, Manikandan Rajarathinam, Neha Kalsekar, Qutaiba Tawfic, Sarah Krause, Derek Nguyen, Eric Liu, Mahesh Nagappa, Yamini Subramani

**Affiliations:** Western University, Schulich School of Medicine and Dentistry, London Health Sciences Centre and St Joseph Health Care, Department of Anesthesia and Perioperative Medicine, London, Ontario, Canada

**Keywords:** Acute pain, Postoperative pain, Magnesium sulphate, Abdominal surgery, Anesthesia, Analgesia

## Abstract

**Background:**

Prior research has established the effectiveness of magnesium in relieving postoperative pain. This article aims to evaluate magnesium sulfate for perioperative analgesia in adults undergoing general abdominal surgery under general anesthesia.

**Objective:**

The primary aim was to assess pain scores at 6 and 24 hours postoperatively in patients receiving magnesium sulfate vs. the control group. Secondary outcomes were postoperative opioid consumption, perioperative complications, and time to rescue analgesia.

**Methods:**

A comprehensive database search identified studies comparing magnesium sulfate with control in adults undergoing general anesthesia for general abdominal surgery. Using random-effects models, data were presented as mean ± Standard Deviation (SD) or Odds Ratios (OR) with corresponding 95% Confidence Intervals (95% CI). A two-sided p-value < 0.05 was considered statistically significant.

**Results:**

In total, 31 studies involving 1762 participants met the inclusion criteria. The magnesium group showed significantly lower postoperative pain scores at both early (within six hours) and late (up to 24 hours) time points compared to the control group. The early mean score was 3.1 ± 1.4 vs. 4.2 ± 2.3, and the late mean score was 2.3 ± 1.1 vs. 2.7 ± 1.5, resulting in an overall Mean Difference (MD) of −0.72; 95% CI −0.99, −0.44; p < 0.00001. The magnesium group was associated with lower rates of postoperative opioid consumption and shivering and had a longer time to first analgesia administration compared to the saline control group.

**Conclusion:**

Magnesium sulfate administration was linked to reduced postoperative pain and opioid consumption following general abdominal surgery.

## Introduction

Effective pain management is a critical objective in both anesthesia and perioperative care. Magnesium modulates pain by inhibiting the N-methyl-D-aspartate receptor, impeding calcium entry into the cell.^1^^,^^2^ Previous clinical trials have affirmed the efficacy of magnesium sulfate in diminishing postoperative pain when juxtaposed with saline control.^1^^,^^3^^,^^4^ While using magnesium sulfate has demonstrated a reduction in postoperative opioid requirements, the optimal dosing regimen remains uncertain.^3^^,^^4^

Prior reviews exploring magnesium as an analgesic adjunct encompassed a spectrum of surgeries characterized by diverse anticipated postoperative pain severities. This systematic review focuses on evaluating the effectiveness of magnesium sulfate for perioperative analgesia in adults undergoing General Anesthesia (GA) for general abdominal surgery and comparing it with an inert control. The primary objective is to assess postoperative pain scores in the group administered magnesium sulfate compared to the control. Secondary outcomes encompass postoperative opioid consumption, intraoperative complications, time to rescue analgesia, and postoperative side effects.

## Methods

This systematic review and meta-analysis followed a predefined protocol, registered with PROSPERO (CRD42022326263), and adhered to PRISMA reporting Guidelines.^5^

### Study selection

We included Randomized Controlled Trials (RCTs) involving Intravenous (IV) magnesium sulfate administration (bolus, infusion, or combination) for perioperative analgesia in adults undergoing general abdominal surgeries under General Anesthesia (GA). Exclusions comprised cohort studies, meta-analyses, systematic reviews, case reports, case series, abstracts, conference proceedings, studies not in English, investigations with non-IV magnesium administration, and those lacking a separate magnesium study arm. Postoperative pain scores (Numerical Rating Scale – NRS) were assessed at early (within 6 hours) and late (up to 24 hours) time points. Side effects and complications related to magnesium sulfate, were recorded in the Operating Room (OR) and/or Postanesthetic Care Unit (PACU). Cumulative postoperative opioid consumption was converted to Morphine Milligram Equivalents (MME) and measured at early and late time points.

### Search strategy

A comprehensive search across PubMed, Medline, Embase, Web of Science, and Cochrane databases used the National Center for Biotechnology Information Medical Subject Headings (NCBI MeSH) descriptors [magnesium (mh) or magnesium (tw) or magnesium sulfate (mh) or magnesium sulfate (tw) or magnesium sulphate (tw)] and [perioperative period (mh) or perioperative (tw) or intraoperative (tw) or intraoperative period (mh) or postoperative (tw) or postoperative period (mh)].

Executed in January 2022, the detailed literature search strategy is in Supplementary Table S1. Two independent authors (YS and NK) scrutinized titles and abstracts, and full texts were assessed by two reviewers (YS and MN). References in the included studies were manually searched. Trials were evaluated for bias using the Cochrane risk of bias tool, graded as “high risk”, “low risk”, or “unclear risk” of bias.^6^^,^^7^ GRADE methodology assessed evidence.^8^

### Data extraction

A standardized protocol guided a data collection form for study characteristics, patient demographics, and intraoperative and postoperative data. Study characteristics were recorded, including the author's name and publication year. Preoperative data, such as age, sex, and Body Mass Index (BMI), were documented. Intraoperative and postoperative data included the type of surgery, duration, doses, and methods of administration of magnesium sulfate and the control, intraoperative complications (such as hypotension and bradycardia), postoperative pain scores (Numerical Rating Scale – NRS), postoperative opioid consumption, time to rescue analgesia, and the incidence of postoperative adverse effects (hypotension, bradycardia, shivering, and Postoperative Nausea and Vomiting [PONV]). Two investigators (YS and YA) extracted data and compiled the results. Any discrepancies in data collection were solved by discussion and dialogue among the team members and by consulting the expert author (MN). Whenever data was missing, we made a concerted effort to contact the corresponding author via email. Our analysis exclusively incorporates studies for which we received a response regarding missing data. YS, NK, and YA ensured data accuracy and completeness.

### Outcome definition

Primary outcome: postoperative pain scores (Numerical Rating Scale – NRS) in magnesium sulfate vs. control groups. Secondary outcomes: postoperative opioid consumption, time to rescue analgesia, intraoperative complications (hypotension, bradycardia), and postoperative side effects (hypotension, bradycardia, shivering, PONV).

### Statistical analysis

Continuous data were presented as mean and standard deviation (SD) and compared as Mean Differences (MD) and 95% Confidence Interval (95% CI); dichotomous data were presented as Odds Ratios (ORs) and 95% CI; *p* < 0.05 was considered significant. A random-effects model accommodated inter-study variation. Egger's test, Begg's test, fail-safe N-test, and funnel plot inspection assessed publication bias. Statistical heterogeneity used the I^2^ statistic and Chi-Square test (I^2^ > 50%, and *p* < 0.05 indicated significant heterogeneity).^9^ Sensitivity and influence analysis was conducted to explore and address heterogeneity by excluding outliers and recalculating pooled estimates. Subgroup and meta-regression analysis was performed to adjust for patient baseline and clinical characteristics to confirm the outcome of the pooled estimate. Review Manager Software (RevMan, V.5.4.1) and comprehensive metanalysis software version 3.0 were used to conduct the analysis.

## Results

**Figure 1:**
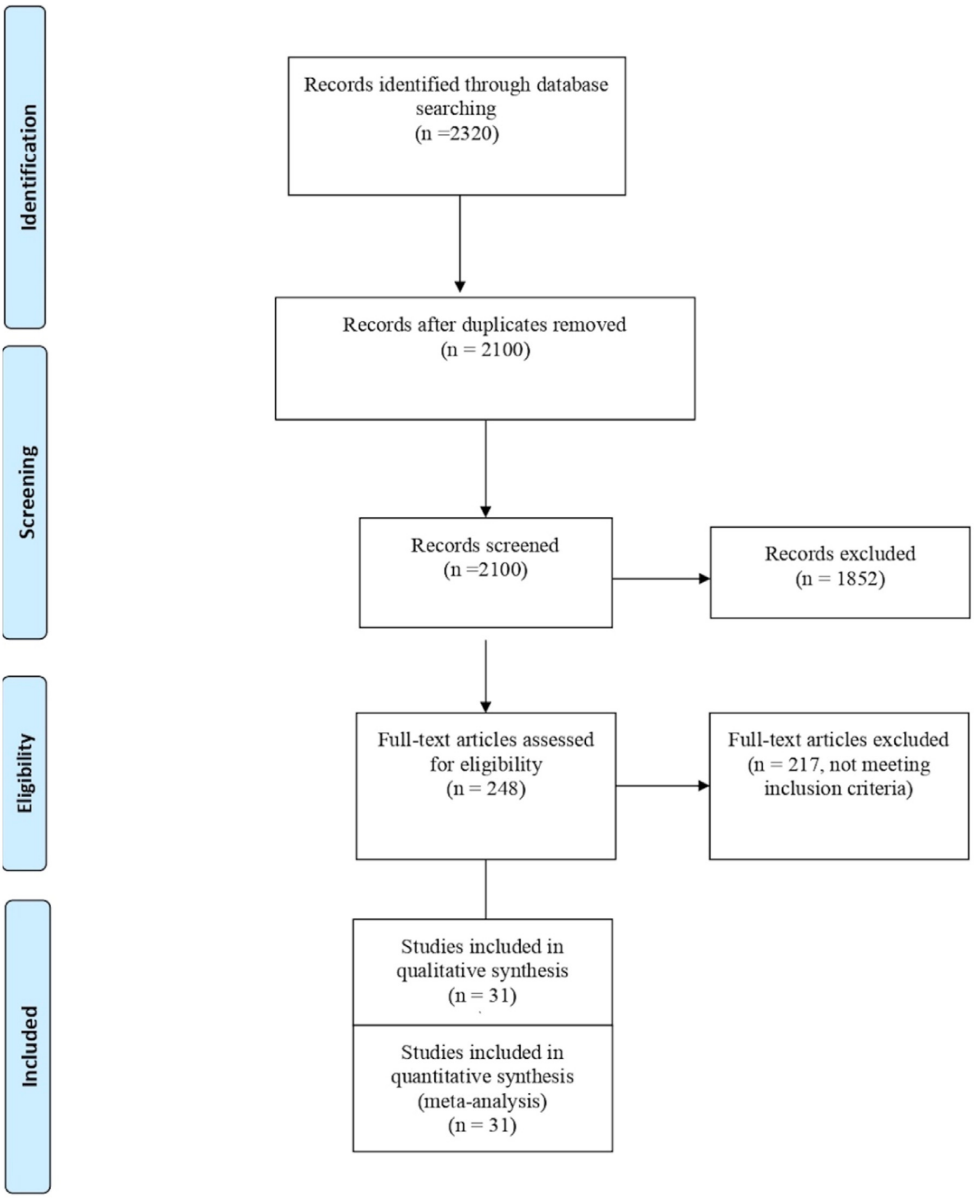
PRISMA flow diagram (n, number).

**Figure 2:**
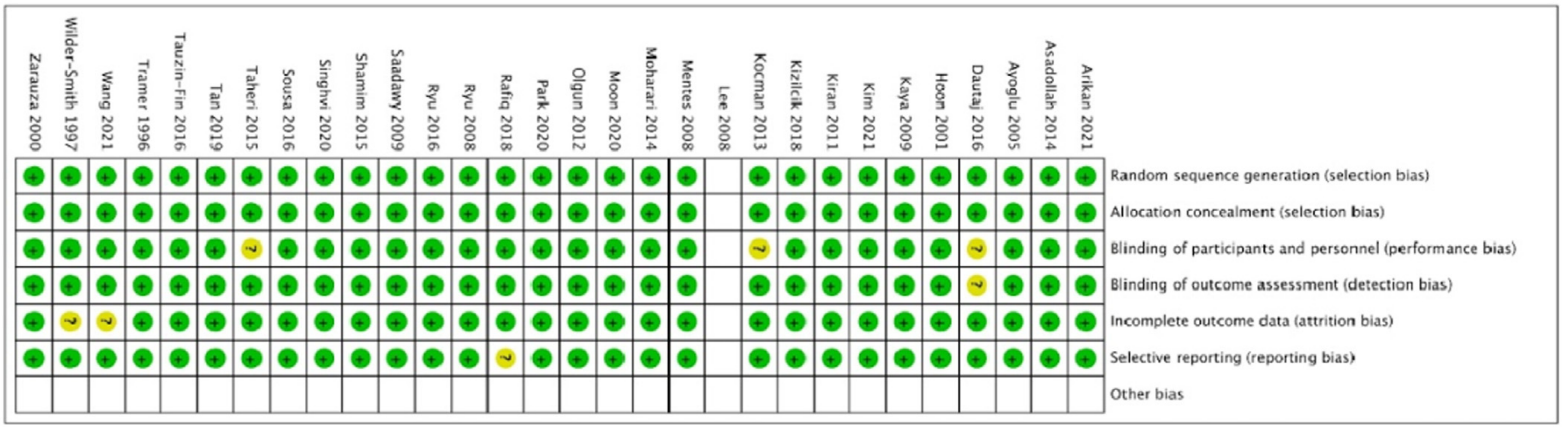
Cochrane risk of bias assessment. Green, Low risk; Yellow, Unclear risk; Red, High risk.

Our initial search identified 2320 studies, which underwent screening by titles and abstracts, resulting in 248 studies for full-text eligibility review. Ultimately, 31 studies with 1762 participants (800 patients in the MgSO_4_ group and 802 patients in the control group) met the inclusion criteria and were included in the analysis (Figure 1).^10^, ^11^, ^12^, ^13^, ^14^, ^15^, ^16^, ^17^, ^18^, ^19^, ^20^, ^21^, ^22^, ^23^, ^24^, ^25^, ^26^, ^27^, ^28^, ^29^, ^30^, ^31^, ^32^, ^33^, ^34^, ^35^, ^36^, ^37^, ^38^, ^39^, ^40^ Participants in the included trials underwent various general abdominal surgeries under General Anesthesia (GA) and received either magnesium sulfate or a control perioperatively. Supplementary Table S2 summarizes the data on the baseline patient characteristics. The quality of the studies was assessed using the Cochrane risk of bias tool, revealing some risks of bias in at least one domain for all studies (Figure 2). The GRADE evidence is summarized in Table 1.

**Table 1:** GRADE Evidence.

Quality assessment									
Outcome	n/ Design/ Sample size	Summary Estimate	Risk of Bias	Magnitude of effect	Inconsistency	Indirectness	Imprecision	Publication Bias?	Quality of Evidence?
Postoperative pain scores									
Early	27/RCT/1525	MD = -1.04	Low	High	No	No	Yes	No	⊕⊕○○ Low
		95% CI: -1.04, -0.55							
Late	23/RCT/1250	MD = -0.41	Low	High	No	No	Yes	No	⊕⊕○○ Low
		95% CI: -0.67, -0.14							
Postoperative opioid consumption									
Early	17/RCT/916	MD = -2.75	Low	High	No	Yes	No	No	⊕○○○ Very Low
		95% CI: -4.20, -1.29							
Late	21/RCT/1907	MD = -8.46	Low	Moderate	No	Yes	No	No	⊕○○○ Very Low
		95% CI: -15.06, -1.87							
Time to rescue analgesia	5/RCT/268	MD = 21.45	Serious	Moderate	No	No	No	No	⊕⊕⊕○ Moderate
		95% CI 6.62, 36.28							
Intraoperative complications	7/RCT/820	OR = 1.27; [0.78, 2.08]	Low	Moderate	No	No	No	No	⊕⊕○○ Low
Postoperative shivering	5/RCT/264	OR = 0.19	Serious	High	Serious	No	No	Yes	⊕⊕○○ Low
		95% CI: 0.09 – 0.44							

RCT, Randomized Controlled Trial; MD, Mean Difference; CI, Confidence Interval; OR, Odds Ratio.

The baseline characteristics were similar between the magnesium and control groups across all individual trials, with no notable variances in patient age (800 vs. 802 patients, 49.4 ± 12.2 vs. 49.4 ± 12.9 years, *p* = 0.93), BMI (215 vs. 218 patients, 28.9 ± 9.3 vs. 28.8 ± 11.4 kg.m^−2^, *p* = 0.57), or surgery duration (697 vs. 696 patients, 111 ± 71.8 vs. 114 ± 70.7 minutes, *p* = 0.36). Fifty-four percent of patients underwent open surgical procedures, while 46% underwent laparoscopic surgeries. The mean magnesium sulfate administered in the studies analyzed in our meta-analysis was 41.1 ± 14.6 mg.kg^−1^ (ranging from 3 to 50 mg.kg^−1^). Sixty-six percent of these studies continued with continuous magnesium infusion following the initial loading dose. The Systematic Review (SR) of the included studies is summarized in Supplementary Table S3.

### Early postoperative pain score

**Figure 3:**
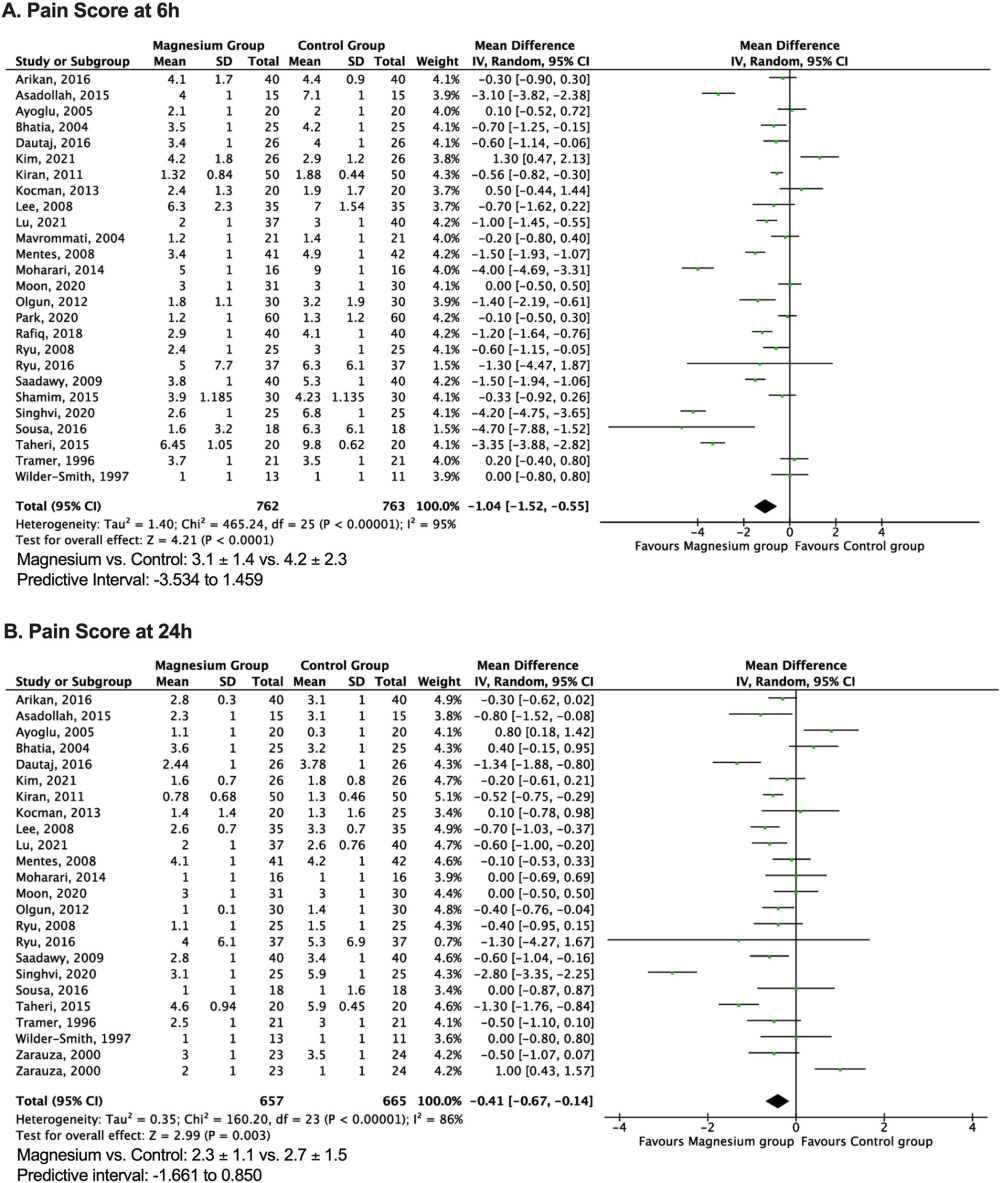
Meta-analysis of early (6 h) and late (24 h) postoperative pain in magnesium and control group patients undergoing surgery. The mean difference of each included study is plotted. Using the random effects model, a pooled estimate of overall mean difference (diamonds) and 95% Confidence Intervals (width of diamonds) summarizes the effect size. CI, Confidence Interval; IV, Inverse Variance.

**Figure 4:**
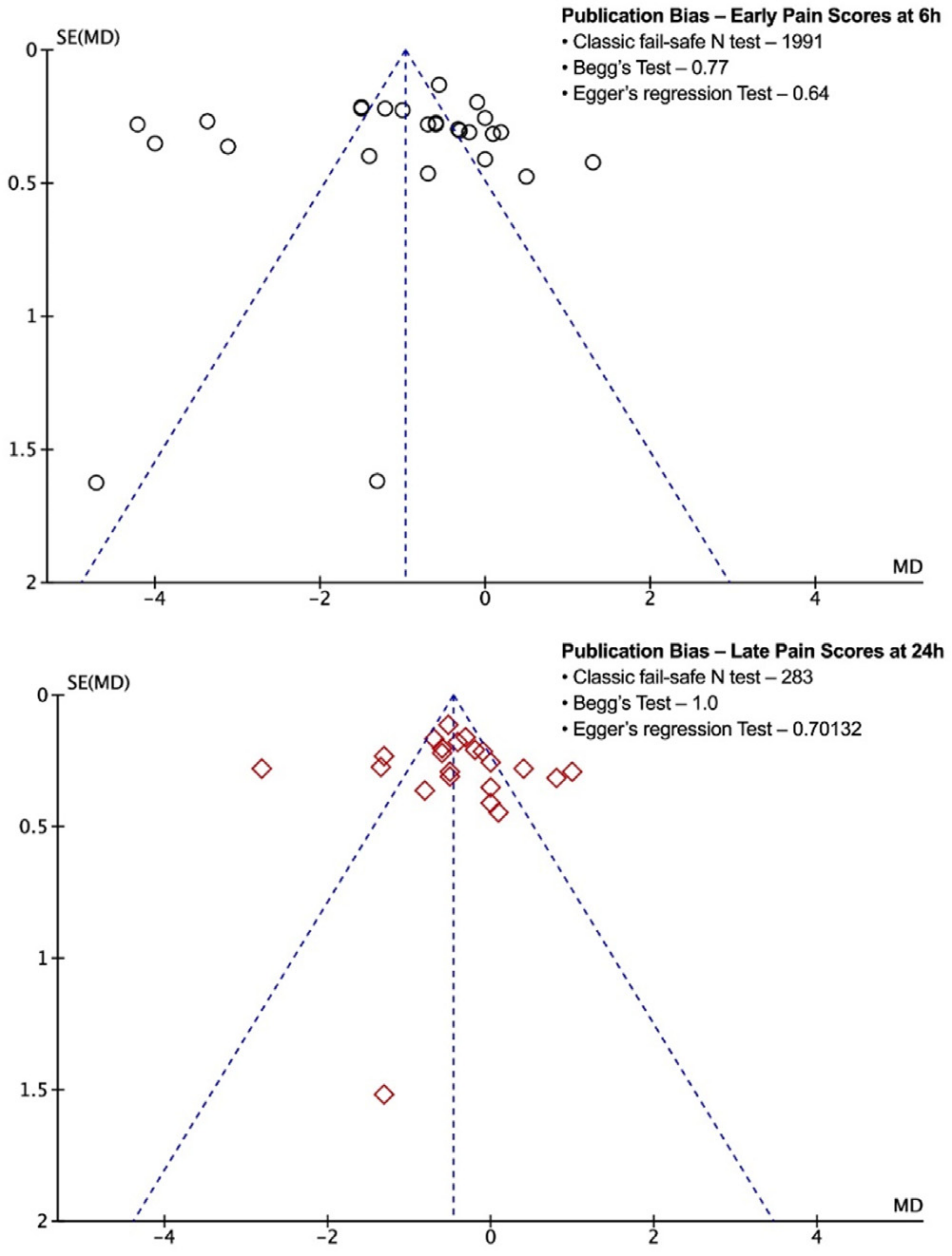
Funnel Plot for the association of early (6 h) and late (24 h) postoperative pain in patients belonging to the magnesium versus the control group. No evidence for substantial publication bias was found in Begg's or Egger's tests. According to classic fail-safe N, missing studies were required to bring the p-value to more than alpha, suggesting the absence of publication bias. SE(MD), Standard Error of Mean Difference.

Twenty-seven trials, consisting of 1525 patients, contributed to the analysis of early (up to 6 hours) postoperative pain scores, indicating a significantly lower mean score in the magnesium group (n = 762, 3.1 ± 2.5) compared to the control group (n = 763, 4.1 ± 2.8) (MD = -1.04; 95% CI: -1.52, -0.55; *p* < 0.0001; I^2^ = 94%) (Figure 3 A).^10^, ^11^, ^12^^,^^14^, ^15^, ^16^, ^17^^,^^19^, ^20^, ^21^, ^22^, ^23^, ^24^, ^25^, ^26^, ^27^, ^28^, ^29^, ^30^, ^31^^,^^33^^,^^34^^,^^36^, ^37^, ^38^, ^39^, ^40^ Publication bias was investigated using a funnel plot (Figure 4), Begg's test (*p* = 0.774), Egger's test (*p* = 0.646), and fail-safe N-test (1991) for each parameter, which was not significant (Figure 4). The funnel plot identified six studies as the major outliers contributing to the heterogeneity. When these studies were excluded and the pooled estimate recalculated, the MD decreased from -1.02 to -0.56, 95%CI narrowed (-0.81, -0.30), and heterogeneity decreased from 95% to 77% without impacting the final inference of our result (*p* < 0.0001).

### Late postoperative pain score

Twenty-three trials, consisting of 1297 patients, were included in the analysis of late (up to 24 hours) postoperative pain scores, revealing a significantly lower mean score in the magnesium group (n = 657, 2.3 ± 2.0) compared to the control group (n = 640, 2.9 ± 2.4) (MD = -0.41; 95% CI: -0.67, -0.14; *p* = 0.006; I^2^ = 87%) (Figure 3 B).^10^, ^11^, ^12^^,^^14^, ^15^, ^16^^,^^19^^,^^20^^,^^22^^,^^24^, ^25^, ^26^, ^27^^,^^29^, ^30^, ^31^^,^^33^^,^^34^^,^^36^, ^37^, ^38^, ^39^, ^40^ No significant publication bias was observed (Figure 4). The funnel plot identified six studies as the major outliers contributing to the heterogeneity. When these studies were excluded and the pooled estimate recalculated, the MD decreased from -0.42 to -0.41, the 95% CI narrowed (-0.53, -0.29), and heterogeneity decreased from 87% to 11% without impacting the final inference of our result (*p* < 0.00001). The overall postoperative pain score data indicated MD = -0.72; 95% CI: -0.99, -0.44; *p* < 0.00001; I^2^ = 93%. Influence analysis confirmed the robustness of the results.

### Early cumulative postoperative opioid consumption

**Figure 5:**
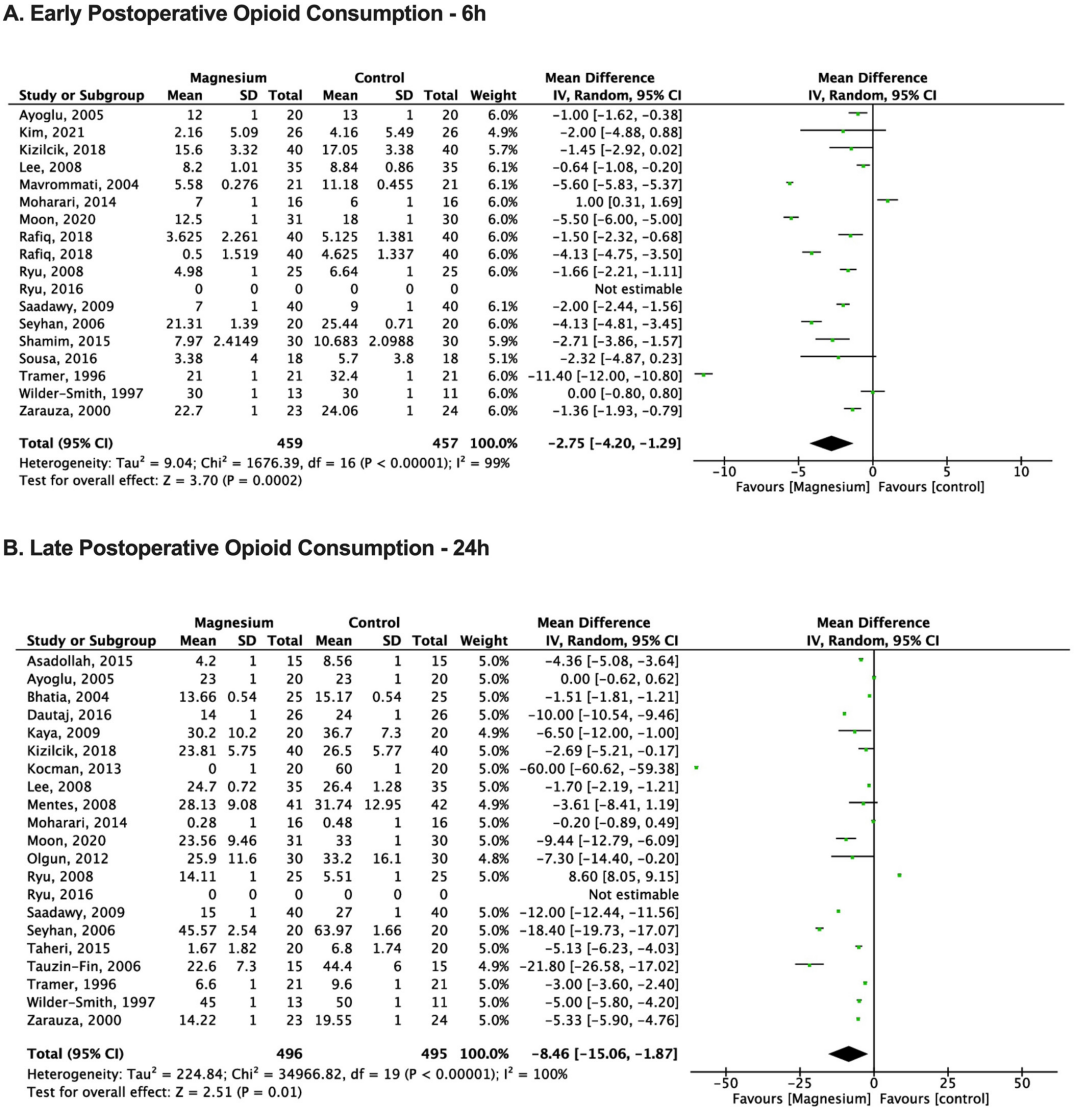
Meta-analysis of early (6 h) and late (24 h) postoperative opioid consumption in magnesium and control group patients undergoing surgery. The mean difference of each included study is plotted. Using the random effects model, a pooled estimate of overall mean difference (diamonds) and 95% Confidence Intervals (width of diamonds) summarizes the effect size. CI, Confidence Interval; IV, Inverse Variance.

Seventeen trials, consisting of 916 patients, contributed to the analysis of early (up to 6 hours) postoperative opioid consumption, demonstrating a significantly lower mean measurement in the magnesium group (n = 459, 9.7 ± 7.7 MME) compared to the control group (n = 457, 12.4 ± 8.4 MME) (MD = -2.75; 95% CI: -4.20, -1.29; *p* = 0.0002; I^2^ = 99%) (Figure 5).^10^, ^11^, ^12^^,^^15^^,^^17^, ^18^, ^19^, ^20^, ^21^, ^22^^,^^25^^,^^26^^,^^28^^,^^30^^,^^32^^,^^34^^,^^38^

### Late cumulative postoperative opioid consumption

Twenty-one trials, consisting of 991 patients, were included in the analysis of late (up to 24 hours) postoperative opioid consumption, indicating a significantly lower mean score in the magnesium group (n = 496, 19.4 ± 12.4 MME) compared to the control group (n = 496, 27.0 ± 16.2 MME) (MD = -8.46; 95% CI: -15.06, -1.87; *p* = 0.01; I^2^ = 100%) (Figure 5).^10^, ^11^, ^12^, ^13^, ^14^^,^^18^, ^19^, ^20^^,^^22^^,^^24^, ^25^, ^26^, ^27^^,^^30^, ^31^, ^32^^,^^35^, ^36^, ^37^, ^38^, ^39^ The overall postoperative opioid consumption data revealed MD = -5.81; 95% CI: -9.09, -2.52; *p* = 0.0005; I^2^ = 100%.

### Time to rescue analgesia

Five trials with 268 patients contributed to the analysis of time to rescue analgesia, indicating significantly longer time in the magnesium group (n = 130, 196.0 ± 315.1 min) compared to the control group (n = 138, 121.0 ± 252.3 min) (MD = 21.45; 95% CI: 6.62, 36.28; *p* = 0.005; I^2^ = 96%) (Supplementary Fig. 1).^13^^,^^15^^,^^17^^,^^21^^,^^30^

### Intraoperative complications

For intraoperative hypotension (n = 375 patients) and bradycardia (n = 445 patients), six^12^^,^^18^^,^^20^^,^^28^^,^^29^^,^^33^ and seven trials,^12^^,^^18^^,^^20^^,^^24^^,^^29^^,^^33^^,^^34^ respectively, were included in the meta-analysis, which showed no significant differences between the magnesium and control groups (OR = 1.52; 95% CI: 0.79–2.92; p = 0.90; I^2^ = 0% and OR = 0.91; 95% CI: 0.38–2.17; *p* = 0.82; I^2^ = 48%, respectively) (Supplementary Fig. 2).

### Postoperative side effects: hypotension, bradycardia, PONV, shivering

Ten (n = 629 patients),^10^^,^^14^^,^^16^^,^^18^^,^^23^^,^^28^^,^^30^^,^^33^^,^^35^^,^^40^ seven (n = 427 patients),^10^^,^^16^^,^^18^^,^^30^^,^^33^^,^^35^^,^^40^ twenty (n = 1134 patients),^10^, ^11^, ^12^, ^13^, ^14^, ^15^, ^16^^,^^19^^,^^20^^,^^22^^,^^23^^,^^25^^,^^27^^,^^29^, ^30^, ^31^^,^^35^^,^^37^^,^^38^^,^^40^ and five trials (n = 264 patients)^12^^,^^22^^,^^30^^,^^34^^,^^37^ respectively were included in the analysis of postoperative hypotension, bradycardia, PONV, and shivering, indicating no significant differences between the magnesium and control groups (OR = 0.90; 95% CI: 0.47–1.72; *p* = 0.75; I^2^ = 0%; OR = 0.91; 95% CI: 0.38–2.17; p = 0.82; I^2^ = 48%; OR = 0.78; 95% CI: 0.59–1.09; *p* = 0.08; I^2^ = 56%, and OR = 0.19; 95% CI: 0.09–0.44; *p* < 0.0001; I^2^ = 0%, respectively) (Supplementary Fig. 3).

### Sensitivity, subgroup and meta-regression analysis

We performed subgroup analysis regarding surgical type (laparotomy vs. laparoscopy: 14 studies vs. 12 studies: MD [95% CI]: -1.20 [-1.34, -1.06] vs. -0.63 [-0.79, -0.46]) and magnesium continuous infusion (yes vs. no: 17 studies vs. 9 studies: -0.85 [-1.43, -0.27] vs. -1.34 [-2.19, -0.48]). No significant differences were observed between these subgroups. In addressing baseline patient and clinical characteristic variations, we conducted a meta-regression analysis, considering factors such as age, gender, BMI, surgical duration, postoperative opioid use, and magnesium dosage as continuous variables. Moreover, we categorized surgical type (laparotomy versus laparoscopy) and magnesium continuous infusion (yes vs. no) to assess their impact on outcomes. Although these variables slightly affected the mean difference, they did not substantially influence the overall estimation of pain scores at 6 and 24 hours (refer to Supplementary Table S3). Additionally, we evaluated the stability of aggregated pain scores through influence analyses by systematically excluding each study from the dataset and recalculating aggregated pain scores based on the remaining studies (see Supplementary Fig. 4A and 4B).

## Discussion

Effective postoperative pain management is crucial as it correlates with adverse clinical outcomes and patient dissatisfaction, potentially leading to chronic pain.^41^ Our findings underscore the beneficial impact of perioperative magnesium sulfate on postoperative pain outcomes, encompassing pain scores, opioid consumption, and adverse effects such as postoperative shivering in adults undergoing General Anesthesia (GA) for general abdominal surgery. Notably, the time to the first analgesic administration was prolonged in the magnesium group compared to the saline control. Importantly, no significant disparities were observed between the two groups concerning intraoperative complications and postoperative side effects, including hypotension, bradycardia, shivering, and Postoperative Nausea and Vomiting (PONV).

This study is distinctive as it represents the first systematic review and meta-analysis consolidating evidence from randomized control trials that specifically focus on the analgesic effects of magnesium sulfate for abdominal surgery. Previous systematic reviews have explored the relationship between magnesium and postoperative analgesia across various surgical disciplines, including orthopedic and cardiac surgery, alongside abdominal surgeries.^1^^,^^3^^,^^42^, ^43^, ^44^ An earlier systematic review and meta-analysis on perioperative intravenous magnesium sulfate administration for postoperative pain also demonstrated a reduction in postoperative opioid consumption compared to control groups, indicating lower postoperative pain. Interestingly, the mode of delivery (bolus, bolus administration, or infusion) did not significantly impact outcomes, and the total magnesium dose administered did not correlate with 24 hour postoperative morphine requirements. Notably, a single bolus administration between 40 and 50 mg.kg^−1^ reduced postoperative morphine consumption.^3^ This aligns with a randomized controlled trial, which suggested that a higher intravenous magnesium dose (50 mg.kg^−1^ bolus + 30 mg.kg^−1^.h^−1^ infusion compared to 30 mg.kg^−1^ + 15 mg.kg^−1^.h^−1^) not only decreased postoperative pain scores but also effectively mitigated pneumoperitoneum-related hemodynamic instability during gastrointestinal laparoscopy.^45^ However, high-dose magnesium administration has been acknowledged to lead to more frequent bradycardia and hypotension likely.^3^ Most trials in our review employed a 40–50 mg.kg^−1^ bolus dose of magnesium.^13^^,^^14^^,^^16^, ^17^, ^18^, ^19^, ^20^, ^21^, ^22^, ^23^, ^24^, ^25^, ^26^, ^27^^,^^30^^,^^33^^,^^34^^,^^37^, ^38^, ^39^, ^40^ Interestingly, two trials in our review utilized much lower magnesium doses, with Kocman et al. prescribing one group at 5 mg.kg^−1^ and another at 7.5 mg.kg^−1^ for laparoscopic cholecystectomy, and Dautaj et al. prescribing 3 mg.kg^−1^ for open cholecystectomy.^31^^,^^36^ Even at lower doses, both trials found that the magnesium groups exhibited significantly better postoperative pain control with no difference in side effects compared to the control group.^31^^,^^36^ Kocman et al. noted that a magnesium dose of 7.5 mg.kg^−1^ was more effective in preventing postoperative pain than the control and magnesium sulfate dose of 5 mg.kg^−1^.^31^ A meta-analysis by De Oliveira et al. also demonstrated that systemic magnesium reduced postoperative pain and opioid consumption after various surgical procedures under GA, with a reduction in postoperative shivering also noted.^42^ Another systematic review by Guo et al. in 2015 confirmed that magnesium administration during GA decreased analgesic consumption and postoperative pain scores without increasing adverse events.^44^ It is worth noting that Lysakowski et al. in 2007 did not agree with the effectiveness of perioperative magnesium on postoperative pain intensity and analgesic requirements.^1^ De Oliveira et al. suggested that this discrepancy could be attributed to the fewer subjects in their observed studies, the inclusion of pediatric populations, and the allowance of various regional anesthesia methods. In contrast, our study was limited to adults and only included those undergoing GA to reduce clinical heterogeneity.^42^

Kawakami et al. conducted a systematic review on magnesium and postoperative shivering, including 64 trials and 4303 patients. They also demonstrated that intravenous magnesium prevented postoperative shivering without resulting in increased adverse events.^46^ The biological mechanism underlying this effect remains uncertain. Shivering can persist despite efforts to avoid hypothermia and may occur even in normothermic conditions. The resulting increase in oxygen demand, leading to heightened carbon dioxide production, may induce myocardial ischemia.^46^ As magnesium exerts a calcium inhibitory effect, causing central arteriolar vasodilation and inducing the production of vasodilator prostaglandins, it possesses anticonvulsant properties and may slightly lower the shivering threshold in patients.^47^^,^^48^

Magnesium sulfate could be an effective adjunct for perioperative analgesia in adults undergoing GA for abdominal surgery. However, our results are constrained by the available evidence in published studies and may have happened by chance. We sought to minimize heterogeneity by restricting our inclusion criteria to general abdominal surgery procedures performed under GA. Despite these constraints, there might still be variability in expected pain severities and pain scores across the studies, even within general surgical procedures such as laparotomy versus laparoscopy. Heterogeneity could stem from differences in baseline chronic pain and opioid tolerance within the patient population, variations in specific surgical procedures, and diverse study designs across the included trials. The varied dosing and administration protocols for magnesium sulfate across studies limit the ability to draw definitive conclusions about the optimal regimen for analgesia in abdominal surgery. Future studies exploring the pharmacokinetics and pharmacodynamics of magnesium in more controlled temperature conditions to measure the impact on postoperative analgesia and shivering may offer valuable insights into its mechanism of action, aiding in identifying optimal dosing strategies for clinicians.

## Conclusion

The findings of the present comprehensive analysis support magnesium sulphate's efficacy in providing perioperative analgesia for adults undergoing general abdominal surgery under general anesthesia. The observed reductions in postoperative pain scores, decreased opioid consumption, lower incidence of shivering, and prolonged time to rescue analgesia administration in the magnesium group highlight its potential as a valuable adjunct in pain management strategies. The results suggest incorporating magnesium sulfate into perioperative protocols may improve patient comfort and outcomes following surgery. Further research, including large-scale clinical trials, may be warranted to confirm these findings and elucidate the optimal dosing regimens and administration protocols for maximizing the analgesic benefits of magnesium sulfate in this context.

## Declaration of competing interest

The authors declare no conflicts of interest.

## Appendix Supplementary materials

Image, application 1

## Footnotes

Supplementary material associated with this article can be found in the online version at doi:10.1016/j.bjane.2024.844524.
