# Supplementary material for: Unravelling the analgesic effects of perioperative magnesium in general abdominal surgery: a systematic review and meta-analysis of randomized controlled trials
Source: Braz J Anesthesiol. 2024 Jun 5;74(4):844524. doi: 10.1016/j.bjane.2024.844524 (PMC11233871; doi:10.1016/j.bjane.2024.844524)

**BJAN-D-24-00039_Supplementary Material**

**Supplementary Table 1** Literature Search Strategies.

| **Search Engine** | **Number of articles** |
| --- | --- |
| PubMed (Medline) | 1469 results |
| EMBASE | 823 results |
| Cochrane | 28 results |

Mesh Key Words: (magnesium [mh] OR magnesium[tw] OR magnesium sulfate[mh] OR magnesium sulfate[tw] OR magnesium sulphate[tw]) AND (perioperative period [mh] OR perioperative [tw] OR intraoperative [tw] OR Intraoperative Period [mh] OR postoperative [tw] OR postoperative period [mh]) & (Magnesium sulfate.mp. or exp magnesium sulfate/ or magnesium sulphate.mp.) AND (exp perioperative period/ or perioperative.mp. or intraoperative.mp. or exp intraoperative period/).

Limit to Human.

Limit to Cochrane Library.

Exclude Medline journals.

**Supplementary Table 2** Baseline patient characteristics.

| **Study ID** | **Locatio** | **Study Type** | **Sample size** | | | **Age (Mean), years** | | **Male gender (%)** | | **BMI (Mean, kg.m^-2^)** | |
| --- | --- | --- | --- | --- | --- | --- | --- | --- | --- | --- | --- |
|  |  |  | **Total (n)** | **MgSO_4_ (n)** | **Control (n)** | **MgSO_4_** | **Control** | **MgSO_4_** | **Control** | **MgSO_4_** | **Control** |
| Arikan et al. 2016 | Turkey | RCT | 80 | 40 | 40 | 56.8 | 58.4 | ‒ | ‒ | ‒ | ‒ |
| Kim et al. 2021 | South Korea | RCT | 52 | 26 | 26 | 63.0 | 65.0 | 100 | 100 | 25.2 | 24.9 |
| Lu et al. 2021 | China | RCT | 77 | 37 | 40 | 46.8 | 45.4 | 21.6 | 27.5 | 24.0 | 23.7 |
| Park et al. 2020 | South korea | RCT | 120 | 60 | 60 | 65 | 65 | 85 | 90 | 25.0 | 25.5 |
| Kizilcik et al. 2018 | Turkey | RCT | 80 | 40 | 40 | 40.7 | 38.4 | 45 | 37.5 | 46.4 | 46.7 |
| Ryu et al. 2016 | South Korea | RCT | 74 | 37 | 37 | 52.3 | 55.7 | 54.1 | 78.4 | 24.4 | 23.6 |
| Sousa et al. 2016 | Brazil | RCT | 36 | 18 | 18 | 49.9 | 47.4 | 0 | 0 | ‒ | ‒ |
| Tauzin-Fin et al. 2006 | France | RCT | 30 | 15 | 15 | 68.0 | 63.0 | 100 | 100 | ‒ | ‒ |
| Dautaj et al. 2016 | Albania | RCT | 52 | 26 | 26 | 43.9 | 44.5 | 57.7 | 53.8 | ‒ | ‒ |
| Taheri et al. 2015 | Iran | RCT | 40 | 20 | 20 | 50.4 | 51.8 | 0 | 0 | ‒ | ‒ |
| Kocman et al. 2013 | Croatia | RCT | 40 | 20 | 20 | 52.7 | 56.7 | 20 | 20 | ‒ | ‒ |
| Olgun et al. 2012 | Turkey | RCT | 60 | 30 | 30 | 47.7 | 45.4 | 16.7 | 16.7 | ‒ | ‒ |
| Kiran et al. 2011 | India | RCT | 100 | 50 | 50 | ‒ | ‒ | ‒ | ‒ | ‒ | ‒ |
| Saadawy et al. 2009 | Egypt | RCT | 80 | 40 | 40 | 38.8 | 42.1 | 12.5 | 20 | ‒ | ‒ |
| Kaya et al. 2009 | Turkey | RCT | 40 | 20 | 20 | 50.0 | 50.0 | 0 | 0 | ‒ | ‒ |
| Mentes et al. 2008 | Turkey | RCT | 83 | 41 | 42 | 47.6 | 46.1 | 14.6 | 28.6 | ‒ | ‒ |
| Lee et al. 2008 | South Korea | RCT | 70 | 35 | 35 | 62.1 | 61.4 | 45.7 | 51.4 | ‒ | ‒ |
| Ryu et al. 2008 | South Korea | RCT | 50 | 25 | 25 | 41.1 | 43.7 | 0 | 0 | ‒ | ‒ |
| Seyhan et al. 2006 | Turkey | RCT | 40 | 20 | 20 | 50.6 | 51.2 | 0 | 0 | ‒ | ‒ |
| Mavrommati et al. 2004 | Greece | RCT | 42 | 21 | 21 | 49 | 51 | 71.4 | 61.9 | ‒ | ‒ |
| Bhatia et al. 2004 | India | RCT | 50 | 25 | 25 | 38.9 | 36.7 | 28 | 20 | ‒ | ‒ |
| Zarauza et al. 2000 | Spain | RCT | 47 | 23 | 24 | 60.6 | 58.6 | 69.6 | 66.7 | ‒ | ‒ |
| Tramer et al. 1996 | Switzerland | RCT | 42 | 21 | 21 | 49.0 | 47.0 | 0 | 0 | ‒ | ‒ |
| Wilder-Smith et al. 1997 | Switzerland | RCT | 24 | 13 | 11 | 48.0 | 45.7 | 0 | 0 | ‒ | ‒ |
| Asadollah et al. 2015 | Iran | RCT | 30 | 15 | 15 | 48.6 | 49.1 | 0 | 0 | 27.3 | 26.8 |
| Ayoglu et al. 2005 | Turkey | RCT | 40 | 20 | 20 | 47.4 | 49.1 | 40 | 35 | ‒ | ‒ |
| Moharari et al. 2014 | Iran | RCT | 32 | 16 | 16 | 41.3 | 45.1 | 37.5 | 50 | ‒ | ‒ |
| Moon et al. 2020 | Korea | RCT | 61 | 31 | 30 | 40.3 | 41.9 | 0 | 0 | ‒ | ‒ |
| Rafiq et al. 2018 | India | RCT | 80 | 40 | 40 | 40.7 | 37.8 | 62.5 | 50 | ‒ | ‒ |
| Shamim et al. 2015 | India | RCT | 60 | 30 | 30 | ‒ | ‒ | ‒ | ‒ | ‒ | ‒ |
| Singhvi et al. 2020 | India | RCT | 50 | 25 | 25 | 36.0 | 33.1 | ‒ | ‒ | ‒ | ‒ |
| Total | ‒ | ‒ | 1762 | 880 | 882 | 49 ± 12 | 49 ± 12 | 32.6 | 33.6 | 28 ± 9 | 28 ± 11 |

**Supplementary Table 3** Patient clinical characteristics and systematic review in tabular column.

| **Study ID (First Author, Year, Reference)** | **Surgery Type** | **Postoperative Outcomes** |
| --- | --- | --- |
|  | **Surgical duration & Intervention** |  |
|  | **Intraoperative outcomes** | **MgSO_4_ vs. Control** |
|  | **MgSO_4_ vs. Control** |  |
| Arikan et al. 2016 | • Type of surgery: Open total abdominal hysterectomy for fibroid disease or uterine myomectomy under general anesthesia | • Pain scores: 2h: 6.4 ± 1.6 vs. 6.6 ± 1.9; 6h: 4.1 ± 1.7 vs. 4.4 ± 0.9; 24h: 2.8 ± 0.3 vs. 3.1 ± 1.0 |
|  | • Surgery Duration: 1.57 ± 0.20hr vs. 1.53 ± 0.23hr | • Postoperative opioid consumption (MME): 48h 58.9 ± 6.5 vs. 65.7 ± 8.2 |
|  | • Interventions: n = 40, bolus dose of magnesium (50 mg.kg^-1^), followed by continuous infusion of 10 mg.kg^-1^.h^-1^ vs. n = 40, same bolus dose, followed by continuous infusion of normal saline |  |
|  |  | • Time to rescue analgesia: N/R |
|  |  | • Postoperative complications: Hypotension: 1 (2.5%) vs. 3 (7.5%); Bradycardia: 1 (2.5%) vs. 2 (5%). Shivering: N/R. PONV: 7 (17.5%) vs. 13 (32.5%) |
|  | • Intraoperative complications: Hypotension: N/R; Bradycardia: N/R |  |
|  | • Intraoperative opioid consumption: N/R |  |
| Kim et al. 2021 | • Type of surgery: Robotic radical prostatectomy | • Pain scores: 30 min: 4.2 ± 1.8 vs. 5.4 ± 2.2; 6h: 4.2 ± 1.8 vs. 2.9 ± 1.2; 24h: 1.6 ± 0.7 vs. 1.8 ± 0.8 |
|  | • Surgery Duration: 218 ± 53.3 vs.224.6 ± 59.6 | • Postoperative opioid consumption: Morphine (mg): 0 (0–6.5) vs. 5.5 (0–7)^a^. In PACU: 2.16 ± 5.09 vs. 4.16 ± 5.49 mg. |
|  | • Interventions: n = 26, MgSO_4_ (50 mg.kg^-1^) diluted with 0.9% saline to a total volume of 100 mL over 10 min, followed by continuous infusion of 10 mg.kg^-1^.h^-1^ vs n = 26, an equal volume of 0.9% saline with the same protocol |  |
|  |  | • Time to rescue analgesia: N/R |
|  |  | • Postoperative complications: Hypotension, Bradycardia & PONV: N/R. Shivering: 0 (0%) vs. 2 (7%) |
|  | • Intraoperative complications: Hypotension: N/R; Bradycardia: 3 (10%) vs. 1 (3%) |  |
|  | • Intraoperative opioid consumption: Total dose of remifentanil (ug) = 600 (270–830) vs. Total dose of remifentanil (ug) = 904 (572–1205) |  |
| Lu et al. 2021 | • Type of surgery: Elective general anaesthesia with laparoscopic cholecystectomy. | • Pain scores: POD1: 2.0 (2.0‒2.0) vs. 3.0 (3.0‒3.0)^a^. Mean (SD): 2 ± 1 vs. 3 ± 1. POD2: 2.0 (2.0‒2.0) vs. 2.0 (2.0‒3.0)^a^. Mean (SD): 2 ± 1 vs. 2.6 ± 0.76. |
|  | • Surgery Duration: 57.5 ± 17.35 vs. 58.6 ± 23.7 min. |  |
|  | • Interventions: n = 37, MgSO_4_ (20 mg.kg^-1^), followed by continuous infusion of 20 mg.kg^-1^.h^-1^ vs. n = 40, control group received saline using the same method. | • Postoperative opioid consumption: N/R |
|  |  | • Time to rescue analgesia: N/R |
|  | • Intraoperative complications: Hypotension: 15 (40.5%) vs. 12 (30.0%); Bradycardia: 12 (32.4%) vs. 11 (27.5%) | • Postoperative complications: Hypotension, Bradycardia & Shivering: N/R; PONV: 26 (65%) vs. 11 (29.7%) |
|  | • Intraoperative opioid consumption: Remifentanil dosage (mg/(kg*min) = 0.19 ± 0.05 vs. Remifentanil dosage (mg/(kg*min) = 0.20 ± 0.04 |  |
| Park et al. 2020 | • Type of surgery: Transurethral resection of bladder tumor under general anesthesia | • Pain scores: 1h: 2.3 ± 0.8 vs. 2.3 ± 1.0; 2h: 2.9 ± 1.8 vs. 2.9 ± 2.0; 6h: 1.2 ± 1.0 vs. 1.3 ± 1.2 |
|  | • Surgery Duration: 46 ± 22 vs.49 ± 28 min | • Postoperative opioid consumption: N/R |
|  | • Anesthesia duration = 67 ± 22 vs.70 ± 28 min. | • Time to rescue analgesia: N/R |
|  | • Interventions: n = 60, loading dose of intravenous MgSO_4_ (50 mg.kg^-1^) administered for 15 min, followed by continuous infusion of 15 mg.kg^-1^.h^-1^ vs. n = 60, received normal saline in the same manner | • Postoperative complications: Hypotension: 9 (15%) vs. 7 (12%); Bradycardia & Shivering: N/R; PONV: 1 (2%) vs. 3 (5%) |
|  | • Intraoperative complications: Hypotension & Bradycardia: N/R |  |
|  | • Intraoperative opioid consumption: N/R |  |
| Kizilcik et al. 2018 | • Type of surgery: Sleeve gastrectomy operation | • Pain scores: N/R |
|  | • Surgery Duration: N/R | • Postoperative opioid morphine consumption: 6h: 15.60 ± 3.32 vs. 17.05 ± 3.38; 24h: 23.81 ± 5.75 vs. 26.50 ± 5.77 |
|  | • Interventions: n = 40, bolus dose of MgSO_4_ (30 mg.kg^-1^), postoperative period: MgSO_4_ infusion of 20 mg.kg^-1^ within 24h vs. n = 40, 50 mL of isotonic saline solution for the same period, postoperative period: 50 mL of serum physiologic infusion within 24h | • Time to rescue analgesia: N/R |
|  |  | • Postoperative complications: Hypotension, Bradycardia, Shivering & PONV: N/R |
|  | • Intraoperative complications: Hypotension & Bradycardia: N/R |  |
|  | • Intraoperative opioid consumption: N/R |  |
| Ryu et al. 2016 | • Type of surgery: Elective laparoscopic gastrectomy | • Pain scores: 30 min: Median (IQR): 50 (0–100) vs. 70 (20–100). Mean ± SD: 50 ± 77 vs. 63 ± 61. 24h: Median (IQR): 40 (0–80) vs. 50 (10–100). Mean ± SD: 40 ± 61 vs. 53 ± 69. |
|  | • Surgery Duration: 190 ± 50 vs. 196 ± 53 min. |  |
|  | • Anesthesia time = 230 ± 49 vs. 239 ± 52 min. | • Postoperative opioid consumption: N/R |
|  | • Interventions: n = 37, 50 mg.kg^-1^ of MgSO_4_ in 100 mL of isotonic saline IV over 10 min, followed by continuous infusion of 15 mg.kg^-1^.h^-1^ vs. n = 37, received the same volume of isotonic saline over the same period. | • Time to rescue analgesia: N/R |
|  |  | • Postoperative complications: Hypotension, Bradycardia, & Shivering: N/R; PONV: 5 (13.5%) vs. 6 (16.2%) |
|  | • Intraoperative complications: Hypotension: 8 (21.6%) vs. 7 (18.9%); Bradycardia: 0 vs 0 |  |
|  | • Intraoperative opioid consumption: remifentanil (mg): 1.5 ± 0.6 vs. 1.6 ± 0.6. |  |
| Sousa et al. 2016 | • Type of surgery: Laparoscopic gynecologic oncology surgery | • Pain scores (VAS, 1–100): 1h: Median (IQR): 10.0 (0‒40) vs 30.0 (0‒57.5). Mean ± SD: 16 ± 32 vs. 63 ± 61. 24h: Median (IQR): 0 vs. 10 (0‒20). Mean ± SD: 0 ± 0 vs. 10 ± 16. |
|  | • Surgery Duration: 269.2 ± 86.9 vs. 250.2 ± 68.5 min |  |
|  | • Anesthesia duration = 355 ± 98 vs. 329.5 ± 81 min |  |
|  | • Interventions: n = 18, bolus dose of MgSO_4_ (20 mg.kg^-1^), followed by continuous infusion of 2 mg.kg^-1^.h^-1^ vs. n = 18, IV saline solution 20 mL in bolus followed by saline infusion | • Postoperative opioid consumption: |
|  |  | Morphine consumption in PACU (mg) 3.38 ± 4.0 vs. 5.7 ± 3.8 |
|  | • Intraoperative complications: Hypotension & Bradycardia: N/R | • Time to rescue analgesia: 5 vs. 5 min. |
|  | • Intraoperative opioid consumption: Total remifentanil 0.17 ± 0.07 μg.kg^-1^.min^-1^ vs. Total remifentanil 0.23 ± 0.13 μg.kg^-1^.min^-1^ |  |
| Tauzin-Fin et al. 2006 | • Type of surgery: Radical retropubic prostatectomy with general anaesthesia | • Pain scores: N/R |
|  | • Surgery Duration: 183 ± 33.4 vs. 177 ± 43.3 min. | • Postoperative opioid consumption: Total tramadol/24h (mg): 226 ± 73 vs. 444 ± 60. Morphine: 22.6 ± 7.3 vs. 44.4 ± 6.0 |
|  | • Interventions: n = 15, MgSO_4_ (50 mg.kg^-1^) in 100 mL of isotonic saline solution over 20 min vs. n = 15, received the same volume of saline over the same period |  |
|  |  | • Time to rescue analgesia: 16 ± 5.6h vs. 13.3 ± 3.7h. |
|  | • Intraoperative complications: Hypotension & Bradycardia: N/R | • Postoperative complications: Hypotension, Bradycardia & Shivering: N/R; PONV: 2 (13.3%) vs. 2 (13.3%) |
|  | • Intraoperative opioid consumption: Sufentanil (μg): 177.7 ± 25.8 vs. 183.3 ± 43.2 |  |
| Dautaj et al. 2016 | • Type of surgery: Abdominal surgery (open cholecystectomy) | • Pain scores (VAS): 4h: 3.4/10 vs. 4.0/10. 8h: 3.78/10 vs. 5.28/10. 24h: 2.44/10 vs. 3.78/10 |
|  | • Surgery Duration: N/R | • Postoperative opioid consumption: Morphine 14 ± 1 vs. 24 ± 1 mg |
|  | • Interventions: n = 26, MgSO_4_ (3 mg.kg^-1^) vs. n = 26, received sodium chloride in the same manner. |  |
|  |  | • Time to rescue analgesia: N/R |
|  | • Intraoperative complications: Hypotension & Bradycardia: N/R | • Postoperative complications: Hypotension, Bradycardia, Shivering & PONV: N/R |
|  | • Intraoperative opioid consumption: N/R |  |
| Taheri et al. 2015 | • Type of surgery: Total abdominal hysterectomy | • Pain scores (NRS): 6h: 6.45 ± 1.05 vs. 9.80 ± 0.62. 12h: 5.90 ± 0.79 vs. 7.80 ± 1.11. 24h: 4.60 ± 0.94 vs. 5.90 ± 0.45 |
|  | • Surgery Duration: 1.53 ± 0.23 vs. 1.55 ± 0.20 h |  |
|  | • Interventions: n = 20, MgSO_4_ (50 mg.kg^-1^) in 100 mL of normal saline vs. n = 20, received 100 mL of 0.9% sodium chloride solution at the same times. | • Postoperative opioid consumption (mg): 24h: Pethidine consumption 16.75 ± 18.23 vs. 68.0 ± 17.42. Morphine: 1.67 ± 1.82 vs. 6.80 ± 1.74 |
|  | • Intraoperative complications: Hypotension & Bradycardia: N/R | • Time to rescue analgesia: N/R |
|  |  | • Postoperative complications: Hypotension: 0 vs. 0. Bradycardia & Shivering: N/R. PONV: 0 vs. 0 |
|  | • Intraoperative opioid consumption: N/R |  |
| Kocman et al. 2013 | • Type of surgery: Elective laparoscopic cholecystectomy | • Pain scores (VAS): 3h: 3.1 ± 1.7 vs. 3.8 ± 2.3; 6h: 2.4 ± 1.3 vs. 1.9 ± 1.7; 24h: 1.4 ± 1.4 vs. 1.3 ± 1.6 |
|  | • Surgery Duration: 51.9 ± 14.8 vs. 52.9 ± 13.6 min. | • Postoperative opioid consumption: 24h: Tramadol 0 vs. 600 mg; 24h: Morphine 0 vs. 60 mg |
|  | • Interventions: n = 20, MgSO_4_ (5 mg.kg^-1^) vs. n = 20, MgSO_4_ (7.5 mg.kg^-1^) vs. n = 20, IV saline. |  |
|  |  | • Time to rescue analgesia: N/R |
|  | • Intraoperative complications: Hypotension & Bradycardia: N/R | • Postoperative complications: Hypotension, Bradycardia, & Shivering: N/R. PONV: 9 (22.5%) vs. 5 (25%) |
|  | • Intraoperative opioid consumption: N/R |  |
| Olgun et al. 2012 | • Type of surgery: Laparoscopic cholecystectomy | • Pain scores (VAS): 4h: 1.8 ± 1.1 vs. 3.2 ± 1.9; 8h: 1.4 ± 1.0 vs. 2.3 ± 1.5; 24h: 1.0 ± 0.1 vs. 1.4 ± 1.0 |
|  | • Surgery Duration: 64.1 ± 20.8 vs. 70.6 ± 16.5 min. |  |
|  | • Interventions: n = 30, MgSO_4_ (40 mg.kg^-1^) infusion over 15 min, followed by continuous infusion of 10 mg.kg^-1^.h^-1^ vs n = 30, same volume of isotonic saline | • Postoperative opioid consumption: Morphine (mg) 25.9 ± 11.6 vs. 33.2 ± 16.1 |
|  |  | • Time to rescue analgesia: N/R |
|  | • Intraoperative complications: Hypotension: N/R; Bradycardia: 5 (16.7%) vs. 8 (26.7%) | • Postoperative complications: Hypotension, Bradycardia, Shivering & PONV: N/R |
|  | • Intraoperative opioid consumption: N/R |  |
| Kiran et al. 2011 | • Type of surgery: Inguinal surgery | • Pain scores (VAS): 4h: 1.32 ± .84 vs. 1.88 ± .44; 8h: 2.74 ± 1.43 vs. 3.84 ± 1.46; 24h: 0.78 ± .68 vs. 1.30 ± .46 |
|  | • Surgery Duration: N/R |  |
|  | • Interventions: n = 50, MgSO_4_ (50 mg.kg^-1^) in 250 mL of isotonic sodium chloride solution IV vs. n = 50, same volume of isotonic sodium chloride solution. | • Postoperative opioid consumption: N/R |
|  |  | • Time to rescue analgesia: N/R |
|  |  | • Postoperative complications: Hypotension: 0 vs. 0; Bradycardia: 0 vs. 0; Shivering & PONV: N/R |
|  | • Intraoperative complications: Hypotension: 0 vs. 0; Bradycardia: 0 vs. 0 |  |
|  | • Intraoperative opioid consumption: N/R |  |
| Saadawy et al. 2009 | • Type of surgery: Elective laparoscopic cholecystectomy | • Pain scores (VAS): Shoulder pain: At rest: 2h: 1.5 vs. 1.45; 6h: 1.2 vs. 1.3. At coughing: 2h: 1.7 vs. 2.3; 6h: 1.9 vs. 2.6. Abdominal pain: At rest: 2h: 3.2 vs. 4.7; 6h: 3.8 vs. 5.3; 24h: 2.8 vs. 3.4. At coughing: 2h: 5 vs. 6.2; 6h: 5.6 vs. 7; 24h: 2.8 vs. 3.8 |
|  | • Surgery Duration: 86.2 ± 14.2 vs. 79.5 ± 16.8 min. |  |
|  | • Interventions: n = 40, bolus dose of MgSO_4_ (50 mg.kg^-1^), followed by continuous infusion of 25 mg.kg^-1^.h^-1^ vs n = 40, received a bolus of 25 mL of normal saline, followed by an infusion of normal saline at 50 mL.h^-1^. |  |
|  |  | • Postoperative opioid consumption: Morphine: 2h: 7 ± 1 vs. 9 ± 1 mg; Morphine: 24h: 15 ± 1 vs. 27 ± 1 mg |
|  | • Intraoperative complications: Hypotension & Bradycardia: N/R |  |
|  | • Intraoperative opioid consumption: Total fentanyl (mg) 254 ± 41.3 vs. 323 ± 70.8 | • Time to rescue analgesia: N/R |
|  |  | • Postoperative complications: Hypotension, Bradycardia & Shivering: N/R; PONV: 16 (40%) vs. 17 (42.5%) |
| Kaya et al. 2009 | • Type of surgery: Elective abdominal hysterectomy for benign diseases | • Pain scores: N/R |
|  | • Surgery Duration: 88.8 ± 26.4 vs. 80.8 ± 28 min. | • Postoperative opioid consumption: Total morphine consumption (mg) 30.2 ± 10.2 vs. 36.7 ± 7.3 |
|  | • Interventions: n = 20, bolus dose of MgSO_4_ (30 mg.kg^-1^), followed by continuous infusion of 3.3 mL.h^-1^ (500 mg.h^-1^ magnesium) vs. n = 20, bolus of 0.2 mL.kg^-1^ of saline, followed by continuous infusion of 3.3 mL.h^-1^ (saline). |  |
|  |  | • Time to rescue analgesia: N/R |
|  |  | • Postoperative complications: Hypotension: 3 (15%) vs. 2 (10%); Bradycardia: 4 (20%) vs. 0; Shivering: N/R; PONV: 5 (25%) vs. 7 (35%) |
|  | • Intraoperative complications: Hypotension & Bradycardia: N/R |  |
|  | • Intraoperative opioid consumption: Remifentanil (μg) 1693 ± 682 vs. 1555 ± 548 |  |
| Mentes et al. 2008 | • Type of surgery: Elective laparoscopic cholecystectomy | • Pain scores (VAS 0‒100): At rest: 4h: 34 vs. 49; 8h: 40 vs. 43; 24h: 41 vs. 42. During cough: 4h: 32 vs. 50; 8h: 33 vs. 49; 24h: 40 vs. 41 |
|  | • Surgery Duration: 72.20 ± 21.18 vs. 74.05 ± 22.77 min. |  |
|  | • Interventions: n = 41, MgSO_4_ (50 mg.kg^-1^) in 100 mL of 0.9% normal saline vs. n = 42, received 100 mL of 0.9% normal saline. | • Postoperative opioid consumption (mg): Tramadol: 281.34 ± 90.82 vs. 317.46 ± 129.59; Morphine: 28.13 ± 9.08 vs. 31.74 ± 12.95 |
|  | • Intraoperative complications: Hypotension & bradycardia: N/R | • Time to rescue analgesia: N/R |
|  |  | • Postoperative complications: Hypotension, Bradycardia & Shivering: N/R; PONV: 4 (9.7%) vs. 7 (16%) |
|  | • Intraoperative opioid consumption: N/R |  |
| Lee et al. 2008 | • Type of surgery: Elective major abdominal surgery (Whipple's operation, PPPD, Colectomy with colorectal anastomosis, Colectomy with coloanal anastomosis) | • Pain scores (VAS, 10): Median (IQR) 6h: 6 (5−8) vs. 7 (6−8); Mean (SD) 6h: 6.3 ± 2.3 vs. 7 ± 1.54; Median (IQR) 24h: 3 (2−3) vs. 3 (3−4); Mean (SD) 6h: 2.6 ± 0.7 vs. 3.3 ± 0.7. |
|  | • Surgery Duration: 273.6 ± 32.1 vs. 280.4 ± 28.7 min. | • Postoperative opioid consumption: Postoperative Cumulative Injected Volume through PCA Pump (mL). Morphine: 0.4 mg.mL^-1^; 6h: 20.51 ± 2.53 vs. 22.11 ± 2.17 mL; 6h: 8.20 ± 1.01 vs. 8.84 ± 0.86 mg; 24h: 61.77 ± 1.80 vs. 66.00 ± 3.22 mL; 24h: 24.7 ± 0.72 vs. 26.4 ± 1.28 mg |
|  | • Interventions: n = 35, MgSO_4_ 50 mg.kg^-1^) administered as a slow IV bolus over 15 minutes, followed by continuous infusion of 10 mg.kg^-1^.h^-1^ vs. n = 35, same volume of isotonic saline (0.9% normal saline). |  |
|  | • Intraoperative complications: Hypotension & Bradycardia: N/R |  |
|  | • Intraoperative opioid consumption: Time weighted mean remifentanil dose (μg.kg^-1^.min^-1^) 0.26 ± 0.21 vs. 0.30 ± 0.14 | • Time to rescue analgesia: 68.7 ± 5.1 vs. 55.1 ± 7.9 min. |
|  |  | • Postoperative complications: Hypotension: 6 (17.1%) vs. 8 (22.9%); Bradycardia: 6 (17.1%) vs. 10 (28.6%); Shivering: 7 (20%) vs. 16 (45.7%); PONV: 9 (25.7%) vs. 3 (8.6%) |
| Ryu et al. 2008 | • Type of surgery: Total abdominal hysterectomy | • Pain scores (VAS, 0‒100): At Rest: 4h: 24 vs. 30; 24h: 11 vs. 15. At Effort: 4h: 28 vs. 33; 24h: 13 vs. 20 |
|  | • Surgery Duration: 169.2 ± 32.3 vs. 162.6 ± 33.7 min. | • Postoperative opioid consumption: PCA vol (mL): Morphine: 50 mg/60 mL; 0.83 mg.mL^-1^; 4h: 6 vs. 8; 4h: 4.98 mg vs. 6.64 mg; 24h: 17 vs. 23; 24h: 14.11 mg vs. 5.51 mg |
|  | • Interventions: n = 25, MgSO_4_ (50 mg.kg^-1^) in 100 mL of isotonic saline over 10 min followed by continuous infusion of 15 mg.kg^-1^.h^-1^ vs. n = 25, same volume of isotonic saline over the same period. |  |
|  | • Intraoperative complications: Hypotension & Bradycardia: N/R | • Time to rescue analgesia: N/R |
|  | • Intraoperative opioid consumption: Remifentanil (mg kg^-1^ min^-1^) 0.11 ± 0.02 vs 0.12 ± 0.02 | • Postoperative complications: Hypotension & Bradycardia: N/R; Shivering: 1 (4%) vs. 9 (36%); PONV: 10 (40%) vs. 19 (76%) |
| Seyhan et al. 2006 | • Type of surgery: Elective hysterectomy with or without salpingo-oopherectomy through Pfannenstiel incision as the first case of the day. | • Pain scores: N/R |
|  |  | • Postoperative opioid consumption: Morphine: mg.kg^-1^; 4h: 0.29 ± 0.11 vs. 0.35 ± 0.06 mg.kg^-1^; 4h: 21.31 (1.39) vs. 25.44 (0.71) mg; 24h: 0.62 ± 0.20 vs. 0.88 ± 0.14 mg.kg-1; 24h: 45.57 (2.54) vs. 63.97 (1.66) mg |
|  | • Surgery Duration: 117.4 ± 8.7 vs. 120.9 ± 18.3 min. |  |
|  | • Interventions: n = 20, MgSO_4_ (40 mg.kg^-1^) 15 min infusion in a total of 100 mL normal saline, followed by 4h infusion of normal saline after tracheal intubation, n = 20, same as previous except it is a 10 mg.kg^-1^.h^-1^ MgSO_4_ infusion after intubation, n = 20, same as previous except it is a 20 mg.kg^-1^.h^-1^ MgSO_4_ infusion after intubation vs. n = 20, 15 min infusion of 100 mL normal saline, followed by 4h infusion of normal saline after tracheal intubation. |  |
|  |  | • Time to rescue analgesia: N/R |
|  |  | • Postoperative complications: Hypotension: 0 vs. 0; Bradycardia: 0 vs. 0; Shivering: N/R; PONV: N/R |
|  | • Intraoperative complications: Hypotension: 0 vs. 0; Bradycardia: 4 (6.7%) vs. 0 |  |
|  | • Intraoperative opioid consumption: Fentanyl (mg.kg^-1^.h^-1^): 3.93 ± 0.62 vs. 4.02 ± 0.61 |  |
| Mavrommati et al. 2004 | • Type of surgery: Reconstruction of postoperative non-obstructed abdominal hernia. | • Pain scores (VAS): 2h: 1.2 vs. 1.4 |
|  | • Surgery Duration: 75 ± 18 vs. 72 ± 21 min. | • Postoperative opioid consumption: Fentanyl (µg.kg^-1^): 0.81 ± 0.23 vs. 1.72 ± 0.35; 55.08 (2.76) vs. 111.8 (4.55) ug. Morphine: 5.58 (0.276) vs. 11.18 (0.455) mg |
|  | • Interventions: n = 21, bolus of MgSO_4_ (30 mg.kg^-1^), followed by continuous infusion of 6 mg.kg^-1^.h^-1^ vs. n = 21, same volume of isotonic sodium chloride solution intravenously |  |
|  | • Intraoperative complications: Hypotension: 0 vs. 0; Bradycardia: N/R | • Time to rescue analgesia: N/R |
|  |  | • Postoperative complications: Hypotension: 0 vs. 0; Bradycardia, Shivering & PONV: N/R |
|  | • Intraoperative opioid consumption: Fentanyl (µg.kg^-1^): 2.83 ± 0.51 vs. 3.52 ± 0.65 |  |
| Bhatia et al. 2004 | • Type of surgery: Open cholecystectomy | • Pain scores (VAS, 0‒100): pain on coughing: 1h: 37 vs. 45; 6h: 35 vs. 42; 24h: 36 vs. 32 |
|  | • Surgery Duration: 77.3 ± 17.6 vs. 76.2 ± 15.1 min. |  |
|  | • Interventions: n = 25, bolus of MgSO_4_ (50 mg.kg^-1^) in 100 mL of 0.9% normal saline vs. n = 25, received 100 mL of 0.9% normal saline | • Postoperative opioid consumption: Morphine: 13.66 ± 0.54) vs. 15.17 ± 0.54 mg |
|  |  | • Time to rescue analgesia: N/R |
|  | • Intraoperative complications: Hypotension & Bradycardia: N/R | • Postoperative complications: Hypotension & Bradycardia: N/R; Shivering: 0 vs. 4 (16%); PONV: 3 (12%) vs. 4 (16%) |
|  | • Intraoperative opioid consumption: morphine requirement (mg) 7.16 ± 1.89 vs. 6.78 ± 1.59. |  |
| Zarauza et al. 2000 | • Type of surgery: Colorectal surgery | • Pain scores (VAS): 2h: 5 vs. 5; 6h: 3 vs. 3.5; 24h: 2 vs. 1 |
|  | • Surgery Duration: 151 ± 32 vs. 158 ± 44 min. | • Postoperative opioid consumption: Morphine (mg.kg^-1^): 0‒12h: 0.32 vs. 0.32 mg.kg^-1^; 0‒12h: 22.7 vs. 24.06 mg; 12‒24h: 0.2 vs. 0.26 mg.kg^-1^; 12‒24h: 14.22 vs. 19.55 mg |
|  | • Interventions: n = 23, dose of MgSO_4_ (30 mg.kg^-1^) in 100 mL of saline, followed by continuous infusion of 10 mg.kg^-1^.h^-1^ over 20h in 500 mL of saline vs. n = 24, saline of same volumes using same procedure |  |
|  |  | • Time to rescue analgesia: N/R |
|  |  | • Postoperative complications: Hypotension: 1 (4.3%) vs. 2 (8.3%); Bradycardia: 0 vs. 0; Shivering: N/R; PONV: 5 (21.7%) vs. 10 (41.7%) |
|  | • Intraoperative complications: Hypotension & Bradycardia N/R |  |
|  | • Intraoperative opioid consumption: fentanyl (ug) 465 ± 83.4 vs. 520.7 ± 72.1 |  |
| Tramer et al. 1996 | • Type of surgery: Elective abdominal hysterectomy | • Pain scores (VAS, 0‒100): At rest: 6h: 37 vs. 35; 24h: 25 vs. 30. During Peak Flow: 6h: 55 vs. 45; 24h: 40 vs. 38 |
|  | • Surgery Duration: 88 ± 21 vs. 108 ± 34 min. |  |
|  | • Interventions: n = 21, 15 mL 20% MgSO_4_, followed by a continuous infusion of 2.5 mL.h^-1^ during 20h vs. n = 21, same volume of saline. | • Postoperative opioid consumption: Morphine with PCA (mg.h^-1^): 0‒6h: 3.5 vs. 5.4 mg.h^-1^; 0‒6h: 21 vs. 32.4 mg; 6‒12h: 1.2 vs. 1.4 mg.h^-1^; 6‒12h: 7.2 vs. 8.4 mg; 12‒24h: 1.1 vs. 1.6 mg.h^-1^; 12‒24h: 6.6 vs. 9.6 mg |
|  | • Intraoperative complications: Hypotension: 1 (4.8%) vs. 0; Bradycardia: 1 (4.8%) vs. 2 (9.5%); |  |
|  |  | • Time to rescue analgesia: N/R |
|  | • Intraoperative opioid consumption: Fentanyl (ug.kg^-1^): 5.8 ± 0.8 vs. 6.3 ± 1.1 | • Postoperative complications: Hypotension & Bradycardia: N/R; Shivering: 0 vs 1 (4.8%); PONV: 6 (28.6%) vs.. 10 (47.6%) |
| Wilder-Smith et al. 1997 | • Type of surgery: Elective abdominal hysterectomy | • Pain scores: 4h: 1 vs. 0; 6h: 1 vs. 1; 24h: 1 vs. 1 |
|  | • Surgery Duration: 62 (52‒74) vs. 54 (50‒72) min. | • Postoperative opioid consumption: Cumulative PCA morphine (mg); 6h: 30 vs. 30; 24h: 45 vs. 50 |
|  | • Interventions: n = 13, 200 mg of magnesium laevulinate as a slow intravenous bolus, followed by continuous infusion of 200 mg.h^-1^ for 5h vs. n = 11, placebo as a slow IV bolus, followed by continuous infusion of placebo. |  |
|  |  | • Time to rescue analgesia: N/R |
|  |  | • Postoperative complications: Hypotension, Bradycardia, & Shivering: N/R; PONV: Nausea: 9 (69.2%) vs. 7 (63.6%); Emesis: 5 (38.5%) vs. 2 (18.2%) |
|  | • Intraoperative complications: Hypotension & Bradycardia: N/R |  |
|  | • Intraoperative opioid consumption: N/R |  |
| Asadollah et al. 2015 | • Type of surgery: Elective lower abdominal laparotomy for hysterectomy and myomectomy. | • Pain scores (VAS 0‒10): 4h: 4 vs. 7.1; 12h: 3.5 vs. 5.6; 24h: 2.3 vs. 3.1 |
|  | • Surgery Duration: 55.2 ± 13.7 vs. 52.5 ± 15.6 min. | • Postoperative opioid consumption: Total consumption of opioid; Pethidine: 42 vs. 85.6 mg; Morphine equivalent: 4.2 vs. 8.56 mg. |
|  | • Interventions: n = 15, MgSO_4_ (50 mg.kg^-1^) in 100 mL of isotonic saline over 10 min, followed by continuous infusion of 8 mg.kg^-1^.hr^-1^ vs. n = 15, receive the same volume of an isotonic saline. |  |
|  |  | • Time to rescue analgesia: N/R |
|  |  | • Postoperative complications: Hypotension, Bradycardia, Shivering & PONV: N/R |
|  | • Intraoperative complications: Hypotension & Bradycardia: N/R |  |
|  | • Intraoperative opioid consumption: N/R |  |
| Ayoglu et al. 2005 | • Type of surgery: Elective laparoscopic cholecystectomy | • Pain scores: Numerical: 4h: 2.1 vs. 2; 8h: 1.2 vs. 1.1; 20h: 1.1 vs. 0.3. Verbal: 4h: 1.05 vs. 1; 8h: 0.3 vs. 0.3; 20h: 0.3 vs. 0.2 |
|  | • Surgery Duration: 58.2 ± 2.8 vs. 61.4 ± 3.7 min. | • Postoperative opioid consumption: Cumulative postoperative morphine consumption (mg): 4h: 12 ± 1 vs. 13 ± 1 mg; 8h: 15 ± 1 vs. 15 ± 1 mg; 20h: 23 ± 1 vs. 23 ± 1 mg |
|  | • Interventions: n = 20, bolus of MgSO_4_ (50 mg.kg^-1^), followed by continuous infusion of 8 mg.kg^-1^.hr^-1^ for the next 4h vs n = 20, received saline bolus and infusion in the same volume. |  |
|  |  | • Time to rescue analgesia: N/R |
|  |  | • Postoperative complications: Hypotension, Bradycardia & Shivering: N/R; PONV: 5 (25%) vs. 8 (40%) |
|  | • Intraoperative complications: Hypotension & Bradycardia: N/R |  |
|  | • Intraoperative opioid consumption: intraoperative alfentanil consumption (mg) 0.2 ± 0.1 vs. 0.5 ± 0.1 |  |
| Moharari et al. 2014 | • Type of surgery: Major non laparoscopic GI surgeries | • Pain scores: Pain severity was reported as none (0), mild (1‒4), moderate (5‒7), and severe (8‒10). Will be reported in order of none, mild, moderate, severe. 4h: 0, 4, 7, 5 vs. 1, 2, 4, 9. 12h: 1, 3, 9, 3 vs. 2, 1, 4, 9. 24h: 1, 13, 1, 1 vs. 4, 3, 8, 1 |
|  | • Surgery Duration: 180.30 ± 23.22 vs. 173.75 ± 25.17 min. |  |
|  | • Interventions: n = 16, MgSO_4_ (40 mg.kg^-1^) infusion in 100 mL normal saline, followed by continuous infusion of 10 mg.kg^-1^.h^-1^ vs. n = 16, received the same volume of an isotonic saline solution. | • Postoperative opioid consumption: Morphine, mg.h^-1^; 4h: 1.75 vs. 1.50; 4h: total: 7 vs. 6 mg; 24h: 0.07 vs. 0.12; 24h: total: 0.28 vs. 0.48 mg |
|  | • Intraoperative complications: Hypotension & Bradycardia: N/R | • Time to rescue analgesia: N/R |
|  | • Intraoperative opioid consumption: doses of fentanyl, μg.kg^-1^ 1.01 ± 0.61 vs. 1.48 ± 0.58. | • Postoperative complications: Hypotension, Bradycardia, Shivering & PONV: N/R |
| Moon et al. 2020 | • Type of surgery: Elective laparoscopic gynecologic surgery | • Pain scores (NRS): 1h: 4 vs. 5; 6h: 3 vs. 3; 24h: 3 vs. 3 |
|  | • Surgery Duration: 79.8 ± 22.8 vs. 92.8 ± 30 min; anesthesia duration = 115.4 ± 25.2 vs. 128.8 ± 30.6 min. | • Postoperative opioid consumption: Cumulative fentanyl consumption (ug): 6h: 125 vs. 180. Morphine equivalent: 12.5 vs. 18 mg. 24h: 235.6 ± 94.6 vs. 330. Morphine equivalent: 23.56 ± 9.46 vs. 33 mg |
|  | • Interventions: n = 31, 40 mL solution of MgSO_4_ (40 mg.kg^-1^) and isotonic saline is injected at a rate of 120 mL.h^-1^ for 20 min as a bolus, followed by continuous infusion of 40 mL of the same solution at 10 mg.kg^-1^.h^-1^ vs. n = 30, isotonic saline is administered in the same volume and manner. |  |
|  |  | • Time to rescue analgesia: N/R |
|  |  | • Postoperative complications: Hypotension, Bradycardia & Shivering: N/R; PONV: 1h: 5 (17%) vs. 12 (40%); 6h: 3 (10%) vs. 10 (33%); 24h: 2 (7%) vs. 7 (23%); 48h: 1 (3%) vs. 4 (13%) |
|  | • Intraoperative complications: Hypotension & Bradycardia: N/R |  |
|  | • Intraoperative opioid consumption: N/R |  |
| Rafiq et al. 2018 | • Type of surgery: Elective abdominal surgery | • Pain scores (VAS): 4h: 3.1 vs. 3.2; 6h: 2.9 vs. 4.1 |
|  | • Surgery Duration: 70.0 ± 13.40 vs. 78.50 ± 19.09 min. | • Postoperative opioid consumption: 6h: Fentanyl (ug): 36.25 ± 22.61 vs. 51.25±13.81. MME: 3.625 ± 2.261 vs. 5.125 ± 1.381; 6h: IV Tramadol (mg) 5.0 ± 15.19 vs. 46.25 ± 13.37. MME: 0.5 ± 1.519 vs. 4.625 ± 1.337 |
|  | • Interventions: n = 40, MgSO_4_ (40 mg.kg^-1^) in 100 mL of 0.9%; normal saline over 15 minutes vs n = 40, 100 mL of 0.9% normal saline over 15 minutes. |  |
|  | • Intraoperative complications: Hypotension & Bradycardia: N/R | • Time to rescue analgesia: n = 32 vs. n = 40; 144.94 ± 123.22 vs 30.68 ± 24.69 min. |
|  | • Intraoperative opioid consumption: Fentanyl (ug) 118.75 ± 22.44 vs. 173.50 ± 23.04 | • Postoperative complications: Hypotension, Bradycardia, Shivering & PONV: N/R |
| Shamim et al. 2015 | • Type of surgery: Laparoscopic cholecystectomy under general anesthesia with endotracheal intubation. | • Pain scores (VAS, 0‒10): 4h: 4.07 ± 1.015 vs. 4.80 ± 1.126; 6h 3.90 ± 1.185 vs. 4.23 ± 1.135 |
|  | • Surgery Duration: N/R | • Postoperative opioid consumption: Total Tramadol consumption (mg): 79.70 ± 24.149 vs. 106.83 ± 20.988; MME: 7.970 ± 2.4149 vs. 10.683 ± 2.0988 mg |
|  | • Interventions: n = 30, MgSO_4_ (50 mg.kg^-1^) in 250 mL of isotonic 0.9% sodium chloride solution over 15‒20 min vs. n = 30, same volume of isotonic 0.9% sodium chloride solution intravenously over same time period. |  |
|  |  | • Time to rescue analgesia: Time of first dose of Tramadol (minutes): 131.72 ± 140.117 vs. 49.33 ± 93.336 |
|  | • Intraoperative complications: Hypotension & Bradycardia: N/R | • Postoperative complications: Hypotension, Bradycardia, Shivering & PONV: N/R |
|  | • Intraoperative opioid consumption: N/R |  |
| Singhvi et al. 2020 | • Type of surgery: Lower abdominal surgery | • Pain scores (VAS, 0‒10): 4h: 3.2 vs. 6.1; 6h: 2.6 vs. 6.8; 24h: 3.1 vs. 5.9 |
|  | • Surgery Duration: 64.40 + 11.69 vs. 63.20 + 13.18 min. | • Postoperative opioid consumption: N/R |
|  |  | • Time to rescue analgesia: N/R |
|  | • Interventions: n = 25, MgSO_4_ (50 mg.kg^-1^) in 250 mL of isotonic sodium chloride solution vs. n = 25, patients were given 250 mL of isotonic sodium chloride solution. | • Postoperative complications: Hypotension: 0 vs. 0; Bradycardia: 0 vs. 0; Shivering: N/R; PONV: 3 (12%) vs 4 (16%) |
|  | • Intraoperative complications: Hypotension & Bradycardia: N/R |  |
|  | • Intraoperative opioid consumption: N/R |  |

MGSO_4_, Magnesium Sulfate; N/R, Not Reported; Mean (SD), Mean (Standard Deviation); VAS, Visual Analogue Scale; PONV, Postoperative Nausea and Vomiting.

^a^ Median (Interquartile range).

**Supplementary Table 4** Meta-regression analysis of the baseline confounding factors for primary outcome.

| **Variable** | **Coefficient** | **Standard error** | **95% CI** | | ***Z*-value** | **2-sided** |
| --- | --- | --- | --- | --- | --- | --- |
|  |  |  | **Lower** | **Upper** |  | **p-value** |
| Early Postoperative pain |  |  |  |  |  |  |
| Age (24) | -0.0931 | 0.1286 | -0.3453 | 0.159 | -0.72 | 0.4692 |
| Male Gender (19) | 0.0595 | 0.0316 | -0.0024 | 0.1214 | 1.88 | 0.0597 |
| BMI (5) | -0.4518 | 1.8204 | -4.0196 | 3.1161 | -0.25 | 0.8040 |
| Surgical Duration (22) | -0.0798 | 0.0341 | -0.1468 | -0.0129 | -2.34 | 0.0194 |
| Morphine Milligram Equivalent dose (14) | -0.1670 | 0.0991 | -0.3612 | 0.0273 | -1.68 | 0.0921 |
| Magnesium loading Dose (24) | -0.0122 | 0.0186 | -0.0486 | 0.0242 | -0.66 | 0.5099 |
| Surgical type: Open vs. Laparoscopy (26) | 0.7874 | 0.4981 | -0.1889 | 1.7637 | 1.58 | 0.1139 |
| Magnesium Continuous infusion: Yes vs. No (26) | -0.4818 | 0.5160 | -1.4931 | 0.5295 | -0.93 | 0.3505 |
| Late Postoperative Pain |  |  |  |  |  |  |
| Age (23) | -0.0381 | 0.0696 | -0.1746 | 0.0983 | -0.55 | 0.5840 |
| Male Gender (18) | 0.0079 | 0.0185 | -0.0284 | 0.0442 | 0.43 | 0.6704 |
| BMI (4) | -1.9143 | 1.6761 | -5.1995 | 1.3708 | -1.14 | 0.2534 |
| Surgical time (20) | -0.0022 | 0.0218 | -0.0449 | 0.0405 | -0.10 | 0.9199 |
| Morphine Milligram Equivalent dose (17) | -0.0042 | 0.0120 | -0.0276 | 0.0193 | -0.35 | 0.7256 |
| Magnesium loading Dose (15) | -0.0141 | 0.0130 | -0.0396 | 0.0115 | -1.08 | 0.2800 |
| Surgical type: Open vs. Laparoscopy (17) | 0.3123 | 0.3098 | -0.2950 | 0.9196 | 1.01 | 0.3135 |
| Magnesium Continuous infusion: Yes vs. No (15) | 0.1286 | 0.3840 | -0.6239 | 0.8812 | 0.33 | 0.7376 |

(n) is the number of studies providing the data on the confounding factor; CI, Confidence Interval.

**Supplementary Figure 1** Meta-analysis of time to rescue analgesia in magnesium and control group patients undergoing surgery. The mean difference of each included study is plotted. Using the random effects model, a pooled estimate of overall mean difference (diamonds) and 95% Confidence Intervals (width of diamonds) summarizes the effect size. CI, Confidence Interval; IV, Inverse Variance.


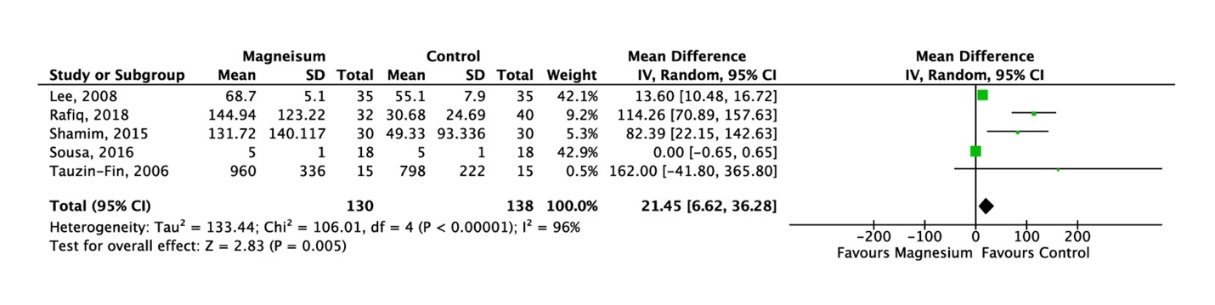


**Supplementary Figure 2** Meta-analysis of intraoperative complications in magnesium and control group patients undergoing surgery. The odds ratio of each included study is plotted. Using the random effects model, a pooled estimate of the overall odds ratio (diamonds) and 95% Confidence Intervals (width of diamonds) summarizes the effect size. CI, Confidence Interval; M-H, Mental-Haenszel.


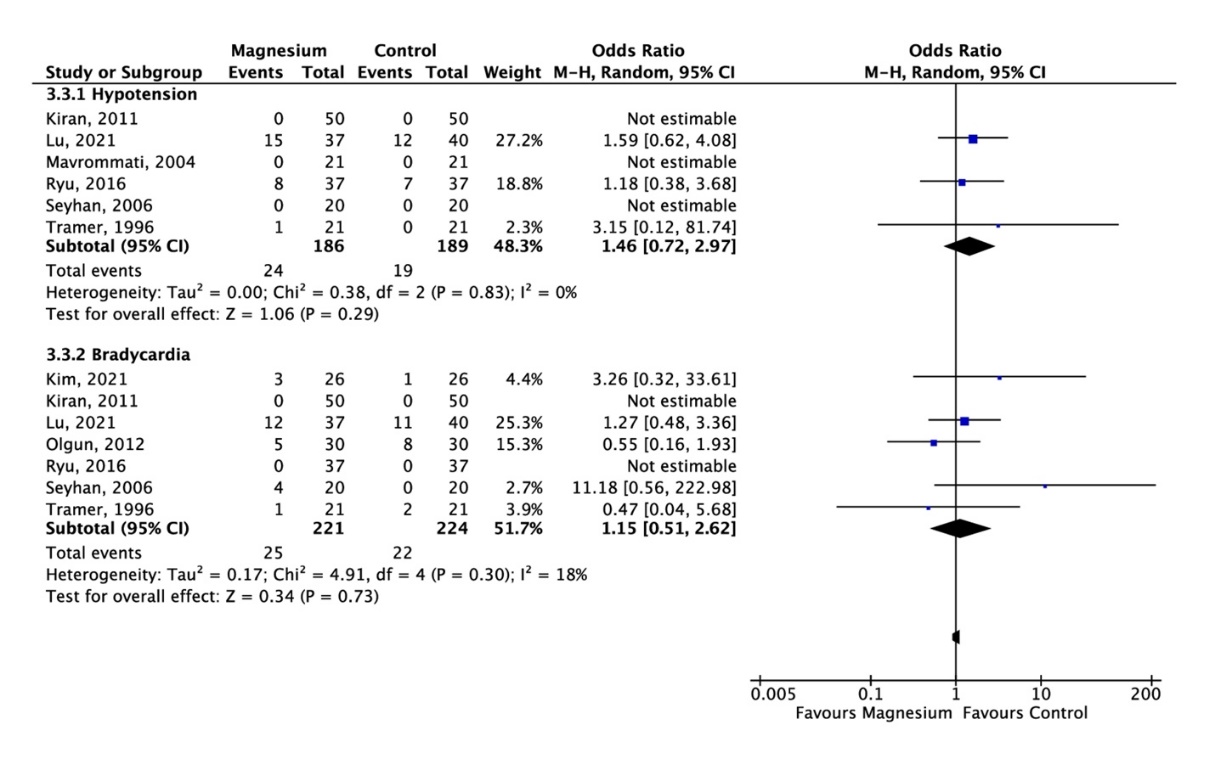


**Supplementary Figure 3** Meta-analysis of postoperative complications in magnesium and control group patients undergoing surgery. The odds ratio of each included study is plotted. Using the random effects model, a pooled estimate of the overall odds ratio (diamonds) and 95% Confidence Intervals (width of diamonds) summarizes the effect size. CI, Confidence Interval; M-H, Mental-Haenszel.


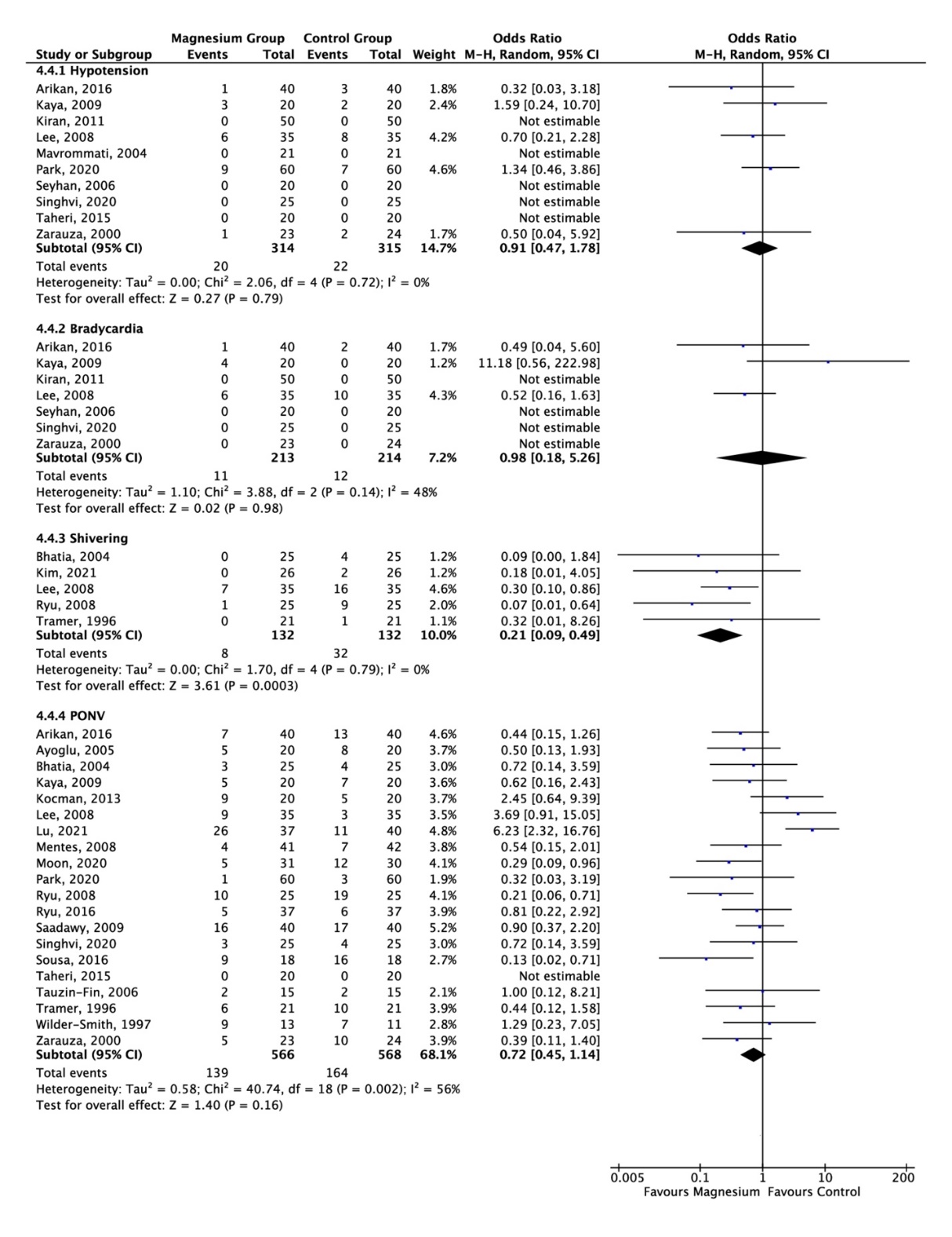


**Supplementary Figure 4** Meta-regression of the effect of baseline patient and clinical characteristics (age, male gender, BMI, time for rescue analgesia, magnesium loading dose, surgery type, and magnesium continuous dose) on the mean difference for pain scores at 6h (4A) and 24h (4B) in the magnesium group versus control patients. Each circle represents a study, telescoped by its weight in the analysis. The relationship was nonsignificant.


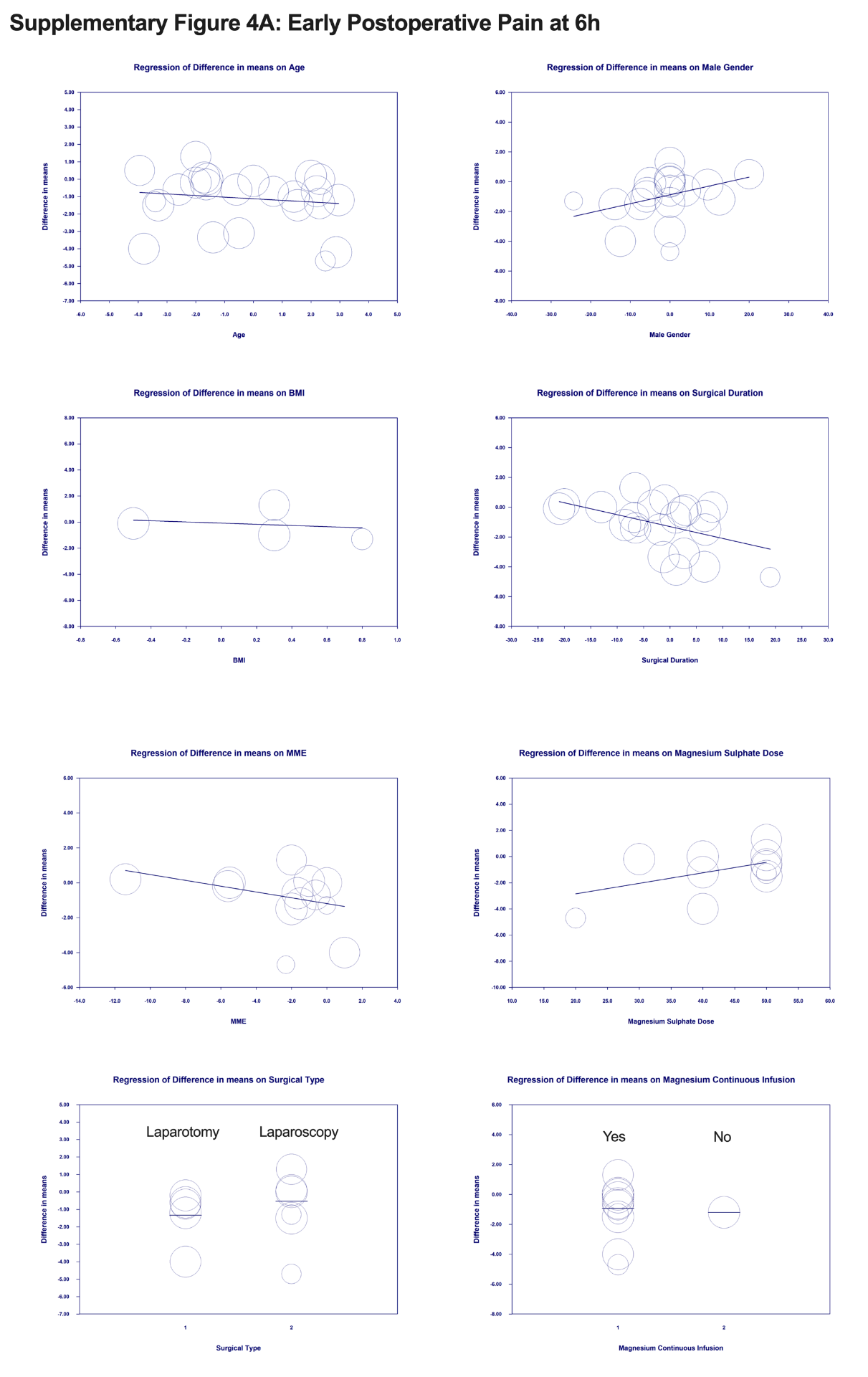

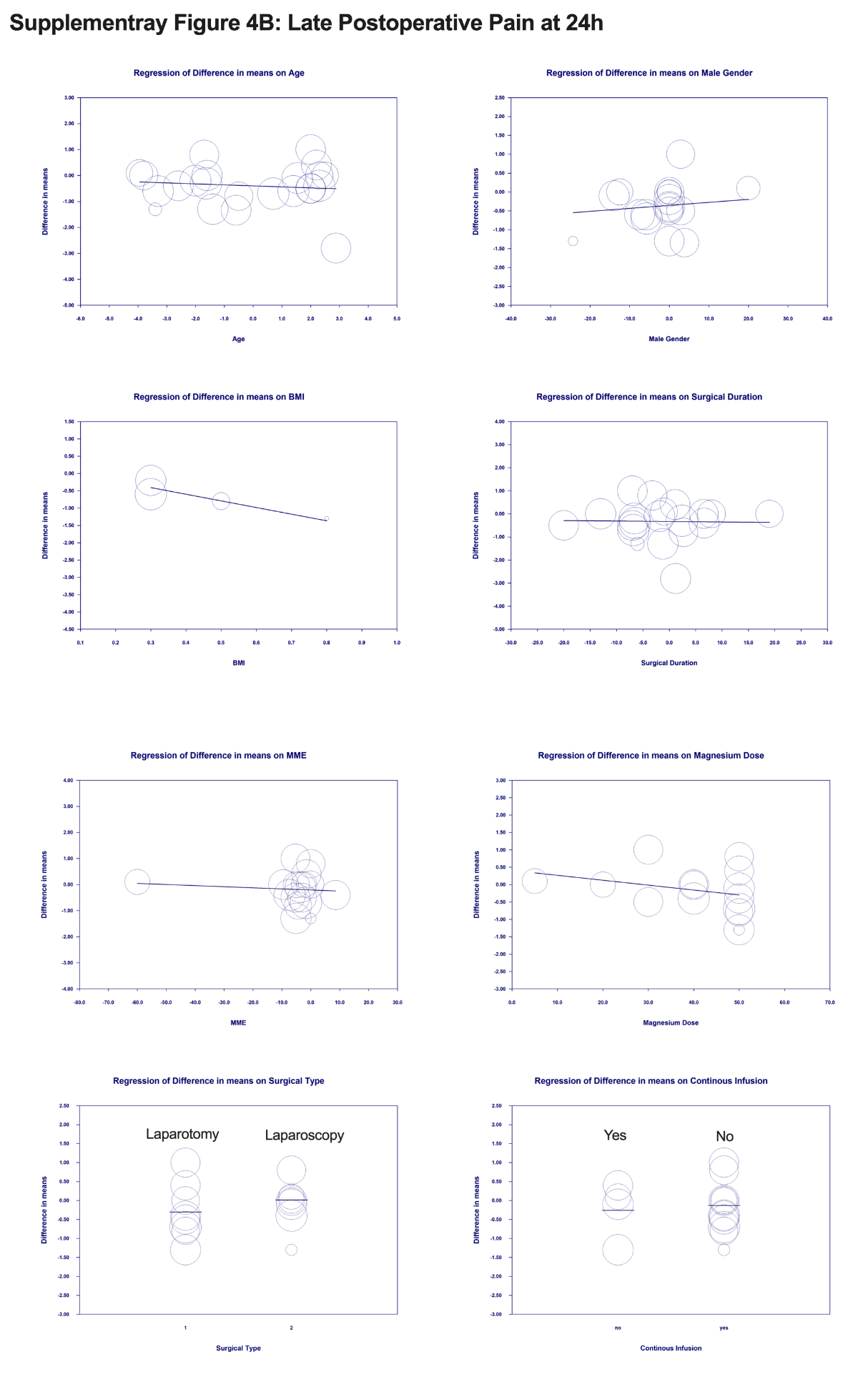

Supplement: Supplementary file 1 [file mmc1.docx]
